# Optimization of and experimentation with a bifurcated swing tube strip fertilizer spreading device based on MBD-DEM coupling

**DOI:** 10.3389/fpls.2024.1456173

**Published:** 2024-10-04

**Authors:** Guoqiang Dun, Quanbao Sheng, Xinxin Ji, Xin Li, Yuhan Wei, Shang Gao, Chaoxia Zhang

**Affiliations:** ^1^ Intelligent Agricultural Machinery Equipment Engineering Laboratory, Harbin Cambridge University, Harbin, China; ^2^ College of Mechanical and Electrical Engineering, Northeast Forestry University, Harbin, China

**Keywords:** bifurcated swing tube, strip fertilizer spreading, uniformity, spatial mechanism, MBD-DEM, coupling simulation

## Abstract

**Introduction:**

To improve the utilization rate of fertilizers, realize the precise spreading of fertilizers in controllable strips, and ensure the uniformity of fertilizer spreading in both longitudinal and transversal directions, a bifurcated swing tube fertilizer spreading device driven by a spatial hammer pendulum crank mechanism was designed.

**Methods:**

First, the drive mechanism was designed based on the cylindrical pair of the mechanism. A mathematical model pendulum equation was used to design the swing tube, and the equation of motion of fertilizer particles was established by analyzing the motion and force of fertilizer particles in fertilizer spreading. The dynamic parameters of the fertilizer spreading device (nozzle height, forward velocity, and swing frequency) were identified as the test factors affecting the uniformity of fertilizer spreading. Second, the coupling model based on MBD-DEM was established, and the coupling simulation analysis of the fertilizer spreading process was carried out using EDEM-RecurDyn software. Taking the nozzle height, forward velocity, and swing frequency as test factors and the uniformity coefficient of longitudinal and transversal fertilizer spreading as evaluation indexes of the fertilizer discharging effect, we analyzed the influence of a single factor on the indexes. Moreover, the ternary quadratic generalized rotating combination response surface test established the regression equations of three factors and two evaluation indexes. Finally, the simulation and bench test were verified under the optimal combination of parameters and compared with the single swing tube bench test with the same parameter conditions.

**Results:**

The results of the single-factor test showed that the fertilizer discharge effect was better when the nozzle height was 350.0–450.0 mm, the forward velocity was 0.5–1.5 m/s, and the swing frequency was 1.40–2.00 Hz. The results of the response surface test proved that the nozzle height, forward velocity, and swing frequency all had a highly significant effect on the uniformity coefficient of fertilizer spreading in the longitudinal and transversal directions (*P*<0.01). Moreover, the optimization concluded that when the nozzle height is 450.0 mm, the forward velocity is 0.5–0.8 m/s, and when the swing frequency is within the range of 1.40–2.00 Hz, the uniformity coefficient of longitudinal fertilizer spreading is ≤25% and the uniformity coefficient of transversal fertilizer spreading is ≤45%. The results of bench validation showed that the errors of longitudinal and transversal fertilizer spreading uniformity coefficients in the bench test were 3.46% and 1.44%, respectively, and the simulation agreed with the bench test. The results of comparative tests showed that the uniformity coefficient of the longitudinal and transversal of the fertilizer spreading device was reduced by 50.33% and 14.95%, respectively, for the bifurcated swing tube compared with that of the single swing tube. It is proved that the bifurcated swing tube strip fertilizer spreading device can achieve the purpose of uniform fertilizer spreading and performs better than the single swing tube in fertilizer spreading.

**Conclusion:**

The results and methods of this study can provide a reference for the design of swing tube strip fertilizer spreading devices and related fertilizer spreading performance tests.

## 1 Introduction

Fertilizer application is the fastest and most effective method of increase crop yields (Wang et al., 2023a; Song et al., 2023a). The reasonable application of chemical fertilizers can improve fertilizer utilization and reduce the amount of fertilizer applied, thus improving economic efficiency, reducing environmental pollution, and promoting sustainable agricultural development (Peng et al., 2023; Zhang et al., 2024; Zhan et al., 2023). Therefore, it is of great practical significance to develop the fertilizer spreading device, precisely control the spreading of fertilizers, and improve the utilization rate and yield of fertilizers while saving costs and increasing benefits.

Presently, the mechanical fertilizer spreading method is mainly divided into strip application and spreading. Relevant scholars at home and abroad have conducted a great deal of research on fertilizer spreading devices for different fertilizer spreading methods. For operations mainly targeting large field acreage, several related scholars have conducted research with centrifugal fertilizer spreading as the primary focus. For example, Zinkevičienė et al. (2021) simulated a centrifugal fertilizer spreader using discrete element software to analyze the effect of its structural parameters on fertilizer spreading performance. Hwang and Nam (2021) used the discrete element method to simulate and analyze the motion of fertilizer particles on a spreader. They optimized the position of the louver holes to improve the spreader’s distribution uniformity. Shi et al. (2018) designed a centrifugal variable-speed fertilizer spreader, evaluated the spreader’s fertilizer spreading performance, and optimized the structural parameters through discrete element simulation tests. Centrifugal fertilizer is suitable mainly for spreading in large areas. It has become a commonly used method of fertilizer application on large farms because it has the advantages of a simple structure and sizeable spreading range. For the complex terrain of mountainous hills and other areas, centrifugal spreading of fertilizer does not adapt to the diversified needs of the land, and many related scholars have conducted research in the form of strip spreading. Dun et al. (2023a) designed a swing tube fertilizer spreading device based on controlling a crank-rocker mechanism with no sharp return characteristic. They simulated the swinging fertilizer spreading process of the swing tube through EDEM software to analyze the effects of the swing tube swinging frequency and the change of the swing tube inclination angle on the longitudinal and transversal fertilizer spreading uniformity. Li and Xia (2013) used the principle of bionics to mimic the fertilizer spreading action of the human arm and designed a general-purpose swing tube fertilizer spreader, which consists of a reciprocating tie rod and a swing tube to form a swing mechanism, and analyzed the motion characteristics of the swing tube to determine the parameters of the whole machine to achieve the uniformity of fertilizer spreading. Zhang (2009) also designed a swing tube fertilizer spreader by simulating the action characteristics of fertilizer spreading by the human arm and designed a swing mechanism consisting of an eccentric disk and a swinging fork, in which the swing tube swings back and forth under the drive of the swing mechanism so that the fertilizers work uniformly through the curved outlet. Cao (2016) designed a swing tube spreading mechanism retrofitted to a rotary tiller and determined the optimal parameters of the mechanism using the variation coefficient of the longitudinal and transversal uniformity of lime spreading as an evaluation index. Bai et al. (2023) designed a lightweight dual-mode automatic variable-speed fertilizer application device and control system for strip and spread fertilizer application. Strip fertilization adopts superimposed swing fertilization and is equipped with Hall sensors to control the rotational speed of the fertilizer discharger to improve fertilization uniformity and accuracy, realizing precise variable-rate strip fertilization. Strip fertilizer spreading has good dynamics and adaptability. The more versatile strip spreading is a good choice when targeting more complex operating environments.

Overall, centrifugal spreading has a sizeable operating width, and the uniformity of fertilizer spreading can be achieved by installing different angles of blades on the spreading disc so that the fertilizer thrown out by each blade is different from the distance to achieve the uniformity of fertilizer spreading (Liu et al., 2017). However, the fertilizer falls into the fertilizer spreading disk, and the distribution among the leaves on the disk is uneven; there are also problems with uneven fertilizer application and low fertilizer utilization (Liu et al., 2023). Moreover, centrifugal fertilizer spreading is mainly suitable for large areas of the operating environment. However, it is unsuitable for some of China’s mountains, hills, and other small pieces of land operating environment. The strip spreading can be limited in the horizontal application of fertilizer. In the fertilization operation, it can be better to maintain longitudinal uniformity, fertilizer near the root, and a higher utilization rate (Dun et al., 2023a; Bai et al., 2023).

To further improve the spreading performance of the strip fertilizer spreader, a bifurcated swing tube strip fertilizer spreading device driven by a spatial hammer pendulum mechanism was designed. The bifurcated structural features separate the discharge of fertilizer flow to reduce the fertilizer due to the phenomenon of swinging overlap and thus uniformly spread to the surface, ensuring the longitudinal and transversal bidirectional uniformity of the spreading. According to the effective use of MBD-DEM coupled simulation for the mechanism motion process at home and abroad (Zhao et al., 2023; Chen et al., 2024; Wang et al., 2024). TIn this paper, a joint technical simulation of the fertilizer spreading process is carried out based on analyzing the kinematic properties of fertilizer particles. To investigate the influence of each parameter on the uniformity of spreading, optimize the parameters and carry out bench tests. The accuracy of the coupled simulation experiment results of the bifurcated swing tube strip fertilizer spreading device is verified by bench and comparison experiments, which provides a reference for designing the swing fertilizer spreading device for granular fertilizers. Thus, it can achieve the purpose of evenly spreading fertilizer, improve the efficiency and quality of fertilizer application, and promote the sustainable development of agriculture.

## 2 Composition and working principle

### 2.1 Structure composition

**Figure 1:**
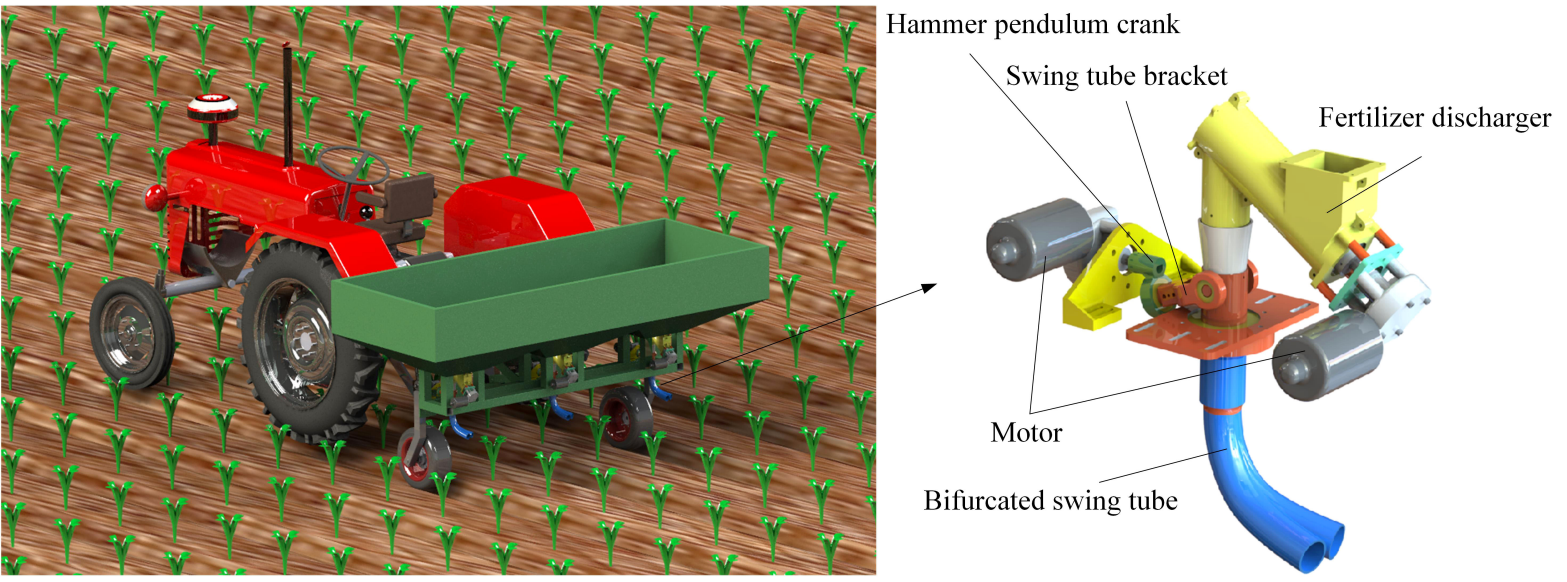
Structure schematic diagram of the bifurcated swing tube strip fertilizer spreading device.

During the growth stage of crop cultivation, small amounts of fertilizer are often required for multiple applications. According to the demand for fertilizer in the growth stage of crops, a bifurcated swing tube strip fertilizer spreading device was designed. The unit can be fitted to sizeable agricultural equipment as a fertilizer applicator unit for Great Plains operations or to small stand-alone applicators for mountainous hill operations. As shown in **Figure 1** as a fertilizer application monolith, the bifurcated swing tube strip fertilizer spreading device mainly consists of a fertilizer discharger, bifurcated swing tube, hammer pendulum crank, swing tube bracket, and motors, which are assembled in the rear of the tractor with a three-point suspension frame for spreading fertilizers.

### 2.2 Working principle

When the bifurcated swing tube strip fertilizer spreading device works, the fertilizer discharged from the fertilizer discharger falls out of the discharge port. Moreover, it enters into the interior of the swing tube. The swing tube bracket is rotationally mated to the bifurcated swing tube using a bearing, and the hammer pendulum crank is fixedly connected to the swing tube bracket. A hammer pendulum crank is fixed to the end of the motor shaft mounted on the frame. During operation, the motor shaft drives the swing tube bracket through the circumferential rotation of the end of the hammer pendulum crank. The swing tube bracket clamps the bifurcated swing tube so that the motor shaft converts the periodic rotation of the hammer crank into a left-right reciprocating swing of the bifurcated swing tube around the swing center. Thus, we realize the function of evenly spreading fertilizer.

**Figure 2:**
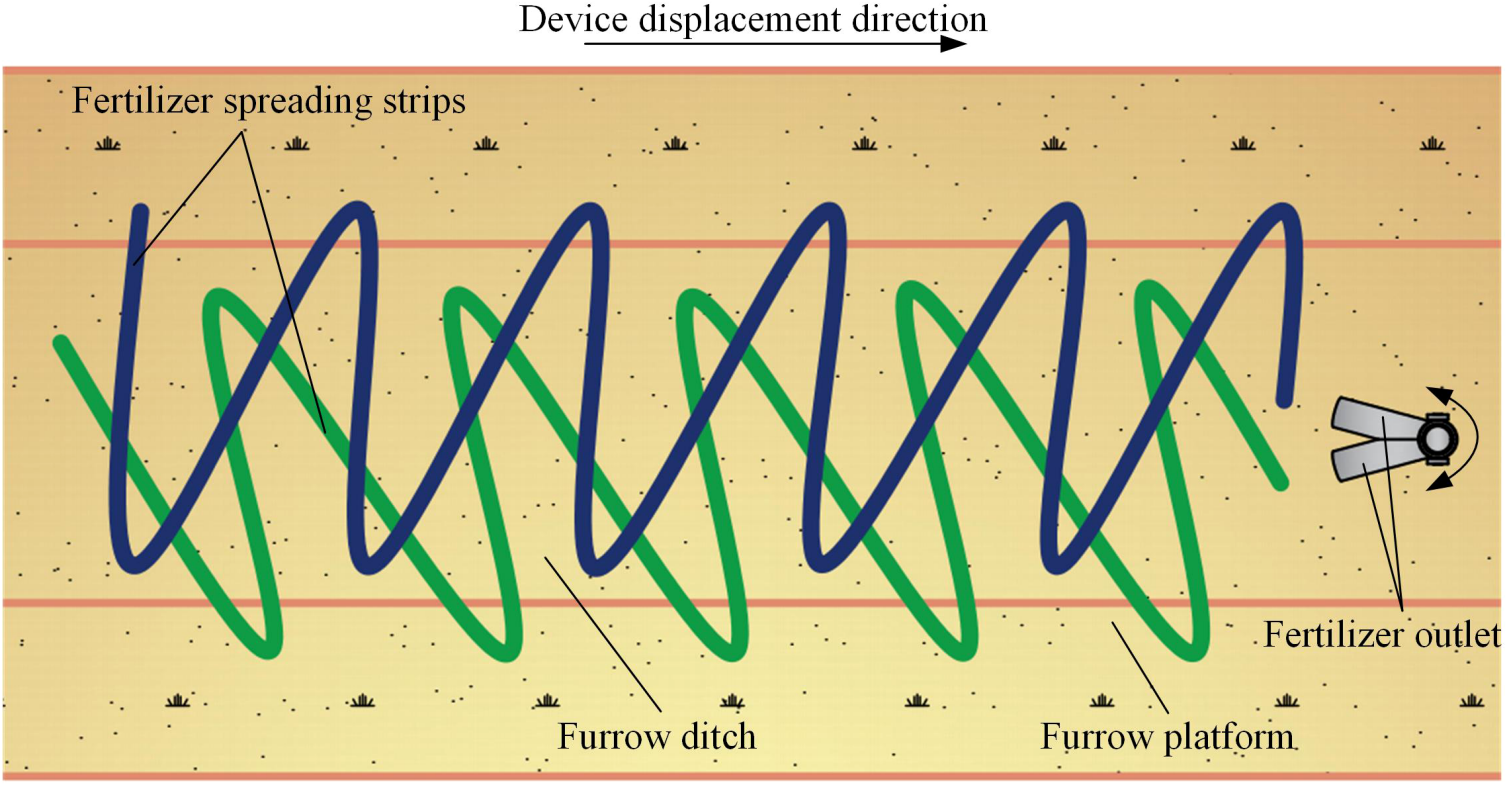
Schematic diagram of fertilizer spreading operation.

The bifurcated swing tube strip fertilizer spreading device spreads fertilizer in the forward direction of the furrow ditch. Under gravity action, the fertilizer discharger will be discharged from the fertilizer tank into the bifurcated swing tube. Finally, the hammer pendulum mechanism drives the bifurcated swing tube to spread the fertilizer particles. The bifurcated swing tube swings the left and right fertilizer outlets simultaneously as the implement moves forward. It spreads the fertilizer evenly to the ground surface cyclically, forming a single-furrow dual-area fertilizer spreading. Forming overlapping complements at the most concave part of the furrow increases the amount of fertilizer applied in the middle while making the fertilizer fall more sparsely on both sides. The device can throw the fertilizer particles into a double fan shape and effectively ensure the uniformity of the fertilizer in the longitudinal and transversal directions within the furrow to uniformly spread fertilizer on the furrow. A schematic diagram of the fertilizer spreading operation is shown in **Figure 2**.

## 3 Mechanism design and motion analysis of the fertilizer particles

### 3.1 Design of the spatial pendulum mechanism

**Figure 3:**
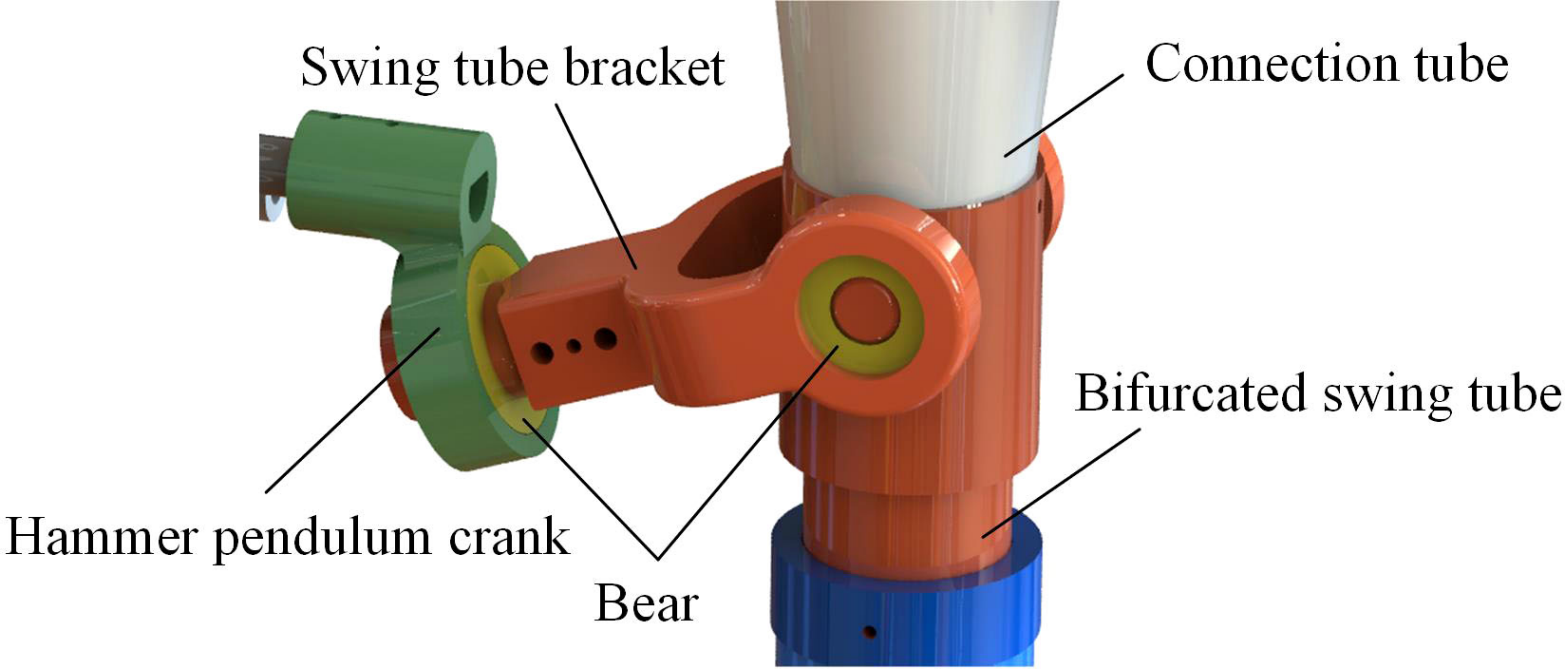
The spatial hammer pendulum crank mechanism.

The bifurcated swing tube uses a spatial hammer pendulum crank mechanism to realize its fertilizer swinging and spreading at the exit of the swing tube. In order to achieve a non-sharp return characteristic for uniformity of fertilizer spreading, the conventional structure of left-right movement of transformation displacement has been changed. Design of space structures using cylindrical pairs. The power input is firstly rotated coaxially in the transverse direction by the cylindrical pair. The power is then transferred to the two cylindrical pairs for power output in up and down, left and right degrees of freedom. The mechanism is shown in **Figure 3**. The three lower pairs cooperate to complete the synthetic motion of torsion and oscillation so that the trajectory of the output end of the mechanism can achieve the effect of reciprocating oscillation motion. The spatial hammer pendulum crank mechanism in this study uses only the cylindrical pair of the lower pair, which possesses the advantages of simple structure, reliable operation, the ability to withstand larger loads, and transmit more power compared than the higher pair (Fang and Yang, 2022).

The mechanism of this study is constructed by a crank, a connecting rod, and a rocker, where the crank *ABC* is the input shaft, *CD*(*D*_0_) is the connecting rod, and the rocker *EOG* is the output shaft. Take the center of the swing tube bracket as the origin, and the *y*-axis coincides with the drive axis to establish a spatial right-angle coordinate system. At the same time, *ABC* and *EOG* are the power input and output that form the rotation axes *A* and *E*, respectively. During operation, *ABC* moves in a fixed axis in space, and owing to the constraint of *CD* (*D*_0_), the cylindrical pair *D* rotates while twisting the cylindrical pair *O*. Thus, *OG* oscillates back and forth in the plane.

To determine whether its motion is reasonable, the degrees of freedom of the spatial mechanism are obtained by subtracting the number of constraints introduced by all the moving parts from the degrees of freedom of the moving parts.


(1) $$\begin{array}{c} F_{dof}=6n-5P_{V}-4P_{IV}-3P_{III}-2P_{II}-P_{I}\text{​​​​}\\ =6\times 3-5\times 1-4\times 3=1 \end{array}$$

where *F_dof_* is the degree of freedom of the spatial mechanism, *n* is the number of movable parts, and *P_I_*, *P_II_*, *P_III_*, *P_IV_*, and *P_V_* respectively, are the number of kinematical pairs of class *I*-*V*.

As the degree of freedom of the overall spatial hammer crank mechanism is one, this mechanism is a single degree of freedom mechanism, and the number of original moving parts is one. The number of original moving parts equals the degree of freedom, and the state of motion is determined.

### 3.2 Kinematic analysis of the pendulum mechanism

**Figure 4:**
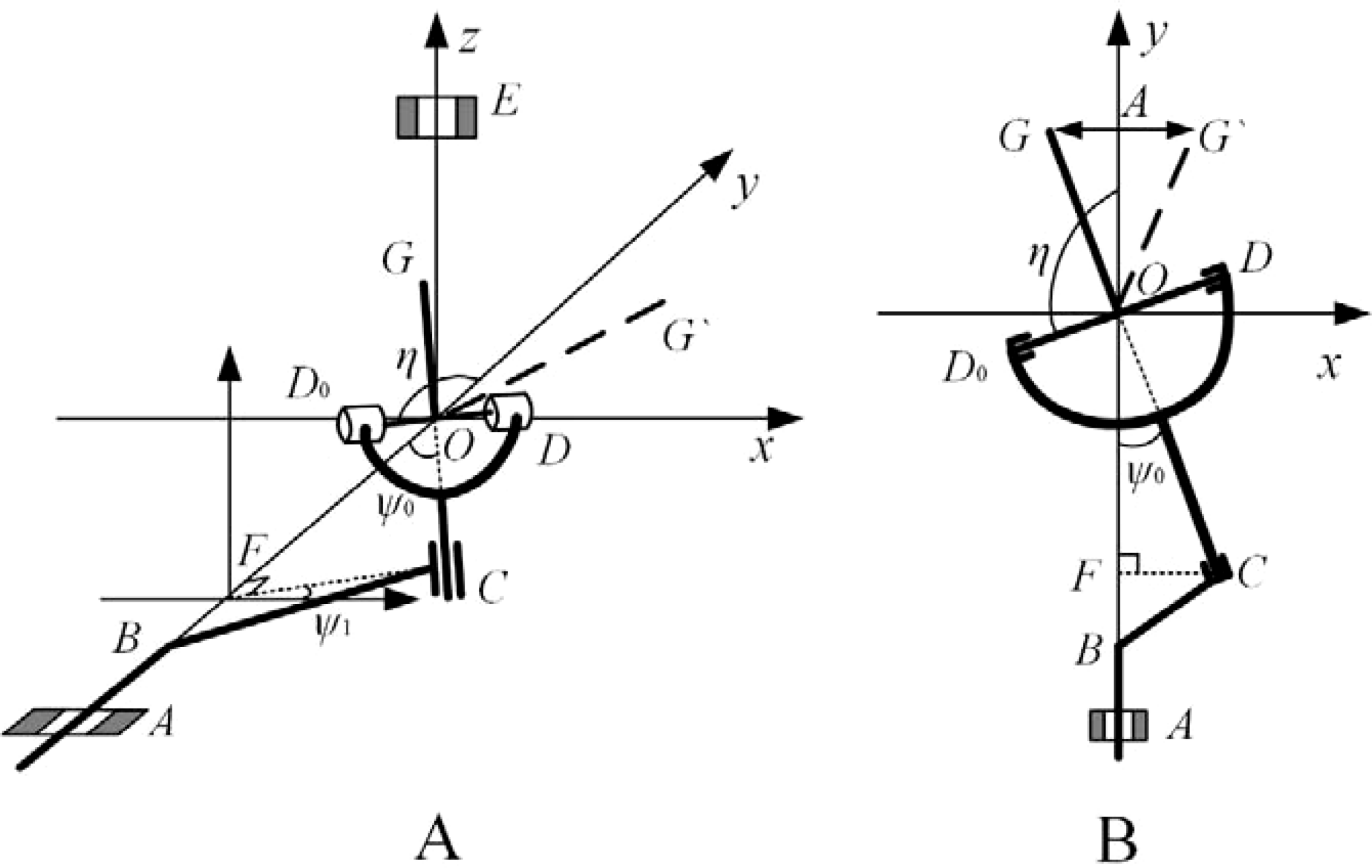
Schematic diagram of the spatial hammer pendulum crank mechanism principle. **(A)** Spatial view. **(B)** Top view.


To determine the motion characteristics and performance parameters of the pendulum mechanism, the angle between the *OC* rod and the *y*-axis is set to *Ψ*_0_, and a vertical line *CF* to the *y*-axis at point *C*. As it rotates in a plane parallel to *xoz*, *CF* is used as the radius of gyration so that the angle between *CF* and the *x*-axis plane is *Ψ*_1_. As the connecting rod *CD* (*D*_0_) oscillates in the *xoy* plane, let *OD*_0_ be at an angle to the *y*-axis. This results in a system of spatial equations for points *C* and *D*_0_, as shown in **Figure 4**.

(2) $$\begin{array}{l} x_{C}=L_{CF}sin\psi_{1}\\ y_{C}=L_{OC}cos\psi_{0}\\ z_{C}=L_{CF}cos\psi_{1} \end{array}\}$$

(3) $$\begin{array}{l} x_{D_{0}}=L_{OD_{0}}sin\eta \\ y_{D_{0}}=L_{OD_{0}}cos\eta \\ z_{D_{0}}=0 \end{array}\}$$

According to the working principle of the spatial pendulum mechanism, the moving range of the two points *D* and *D*_0_ of the connecting rod *CD* (*D*_0_) is always in the *xoy* plane and oscillates with the rotation of *BC*. As *OC* is perpendicular to the *OD* line, there is:


(4) $$\vec{OC}\cdot \vec{OD_{0}}=\left\{x_{C},y_{C},z_{C}\right\}\left\{\begin{array}{l} x_{D_{0}}\\ x_{D_{0}}\\ x_{D_{0}} \end{array}\right\}=x_{C}x_{D_{0}}+y_{C}x_{D_{0}}+z_{C}x_{D_{0}}=0$$

(5) $$L_{CF}sin\psi_{1}\cdot L_{OD_{0}}sin\eta +L_{OC}cos\psi_{0}L_{OD_{0}}cos\eta =0$$

The above equation is collated to give the following equation:


(6) $$tan\eta =-\frac{sin\psi_{0}}{sin\psi_{1}}=-\frac{cos\psi_{0}}{tan\left(\frac{\pi }{2}-\psi_{0}\right)cos\left(\frac{\pi }{2}-\psi_{1}\right)}$$

The first order derivation of Equation 6 is then performed.


(7) $$\dot{\eta }=-\frac{tan\eta tan\left(\frac{\pi }{2}-\psi_{1}\right)}{1+tan^{2}\eta }$$

The second order derivation of Equation 6 is then performed.


(8) $$\ddot{\eta }={\dot{\psi }}_{1}\dot{\eta }tan\left(\frac{\pi }{2}-\psi_{1}\right)-2\dot{\eta }tan\eta +{\dot{\psi }}_{1}^{2}tan\eta \frac{1+\left(\frac{\pi }{2}-\psi_{1}\right)}{1+tan^{2}\eta }$$

When *Ψ*_1_ = -π/2 or π/2, *η =* π/2, $\dot{\eta }=\underline{+}{\dot{\psi }}_{1}\tan\psi_{0}$ , $\ddot{\eta }=0$ , Through the above analysis $\ddot{\eta }=0$ can be seen at this moment for the angle function inflexion point, in the angle *η* = π/2+*Ψ*_0_ and *η* = π/2-*Ψ*_0_ range to achieve the angle of positive and negative conversion, to achieve the spatial pendulum mechanism in the spatial rotation of the movement of the left and right swing motion for the purpose of the power output. Moreover, the design of the horizontal length of the swing tube can be derived from the joint Equations 12 and 13:


(9) $$\begin{array}{c} A=x_{G^{\prime }}-x_{G}=L_{OG}\left(cos\eta_{min}-cos\eta_{max}\right)\\ =L_{OG}\left[cos\left(\psi_{0}-\frac{\pi }{2}\right)-cos\left(\psi_{0}+\frac{\pi }{2}\right)\right] \end{array}$$

Included among these,


(10) $$L_{OG}=\frac{A}{2sin\psi_{0}}$$

where *A* is the range of swing at the end of the swing tube (mm).

**Figure 5:**
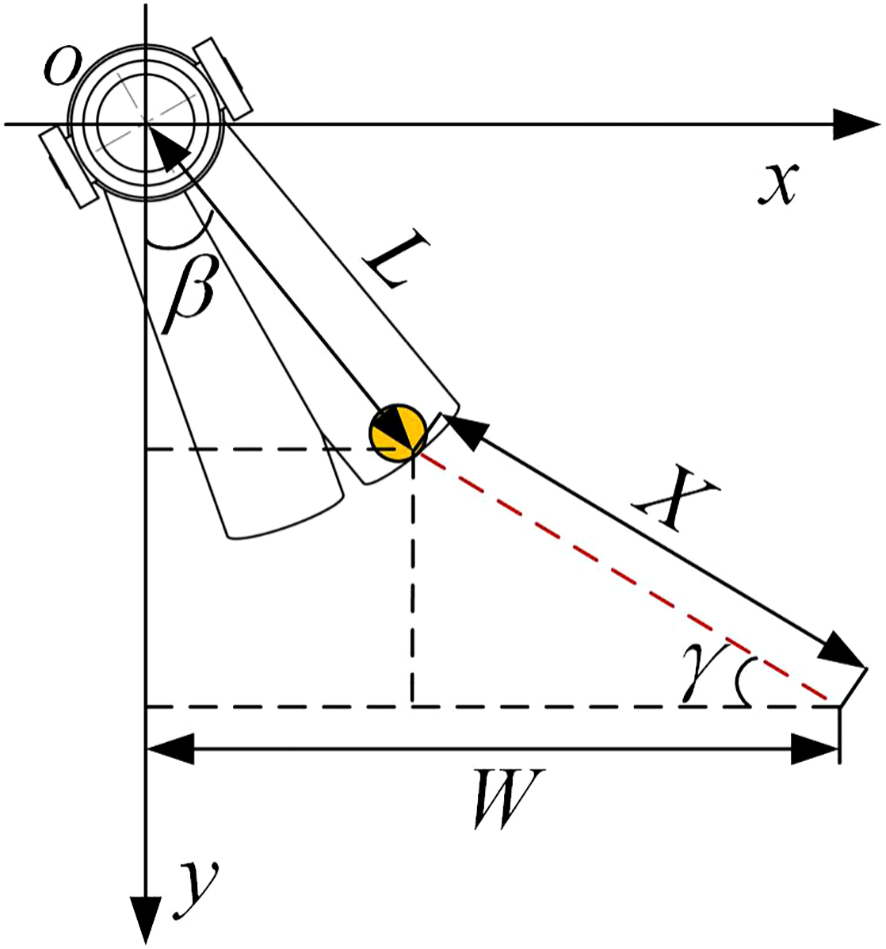
Top view of fertilizer spreading.


The fertilizer spreading top view schematic is shown in **Figure 5**. Take the actual field operation of maize, tiny single rows with a width of approximately 60–70 cm between the rows, as an example. As the bifurcated swing tube fertilizer spreading device only needs to spread fertilizer evenly to the two sides of the ridge and furrow when working, to meet the agronomic requirements of maize planting with a minimum spacing of 60 cm between the ridges, the range *W* of spreading applied at the swing tube mouth shall be satisfied [Equation (11)].

(11) W=Lsinβ+Xcosγ≥300

where *β* is the angle between the unilateral swing tube and the longitudinal direction (°), *γ* is the angle between the fertilizer spreading path and the lateral direction (°), *L* is the horizontal length of the swing tube (mm), and *X* is the distance from the fertilizer throwing plane (mm).

### 3.3 Swing tube design

**Figure 6:**
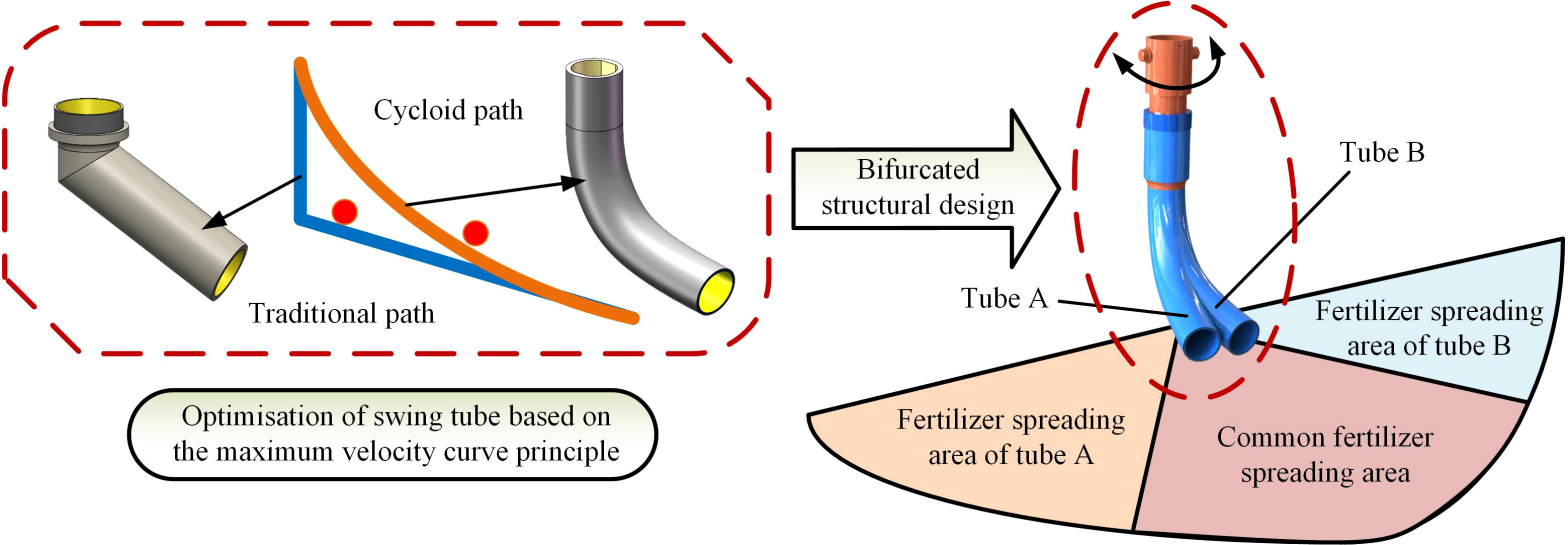
Bifurcated swing tube design schematic.

The fertilizer spreading tube’s structure directly affects the fertilizer application’s effect and the uniformity of its distribution on the ground. Conventional swing tubes are mainly angled for downward application and do not have smooth curved surfaces (Dun et al., 2023a; Li and Xia, 2013; Zhang, 2009; Bai et al., 2023). This structure will lead to slow fertilizer discharge or fertilizer congestion under certain circumstances; therefore, the design of the swing tube shape using the maximum velocity curve principle can be better than the straight line path under the exact height of the fertilizer discharge effect in the shortest possible time to discharge the fertilizer in the swing tube (Wu et al., 2023; Yang et al., 2024). In addition to the fertilizer path in the swing tube, the structure of the swing tube also influences the fertilizer spreading effect. The traditional swing tube spreading device is usually a single tube implementation; its structure is simple and easy to implement. However in fertilizer application, it has a giant swing and causes a more concentrated fertilizer drop point. In response to this phenomenon, a bifurcated swing tube structure was designed with two fertilizer-spreading tubes protruding from one side in the same drop tube, as shown in **Figure 6**.

The A and B fertilizer divider tubes are placed at an angle to each other, and the fertilizer flows out of the main tube. Driven by the spatial hammer crank mechanism, the single tube swings in a double area within the spreading range and has an intersection and overlap in the middle of the area so that the total amount of fertilizer can be spread in diverted strips. A lower limit of fertilizer spreading width of 60 cm was used as the basis for the design (He et al., 2021; Zhao et al., 2021a; Gou et al., 2022) and was affected by the combined effect of the size of the machine, the material used for processing, and the comfort of use. Main working parameters of the bifurcated swing tube device are shown in **Table 1**.

**Table 1:** Main working parameters of the bifurcated swing tube device.

Parametric	Value
Swing tube outer diameter/mm	35
Swing tube inner diameter/mm	30
Swing tube bifurcation angle/°	20
Swing tube actual length/mm	190
Crank length/mm	29
Connecting rod length/mm	79.5
Swing range/°	40

### 3.4 Fertilizer spreading force analysis

**Figure 7:**
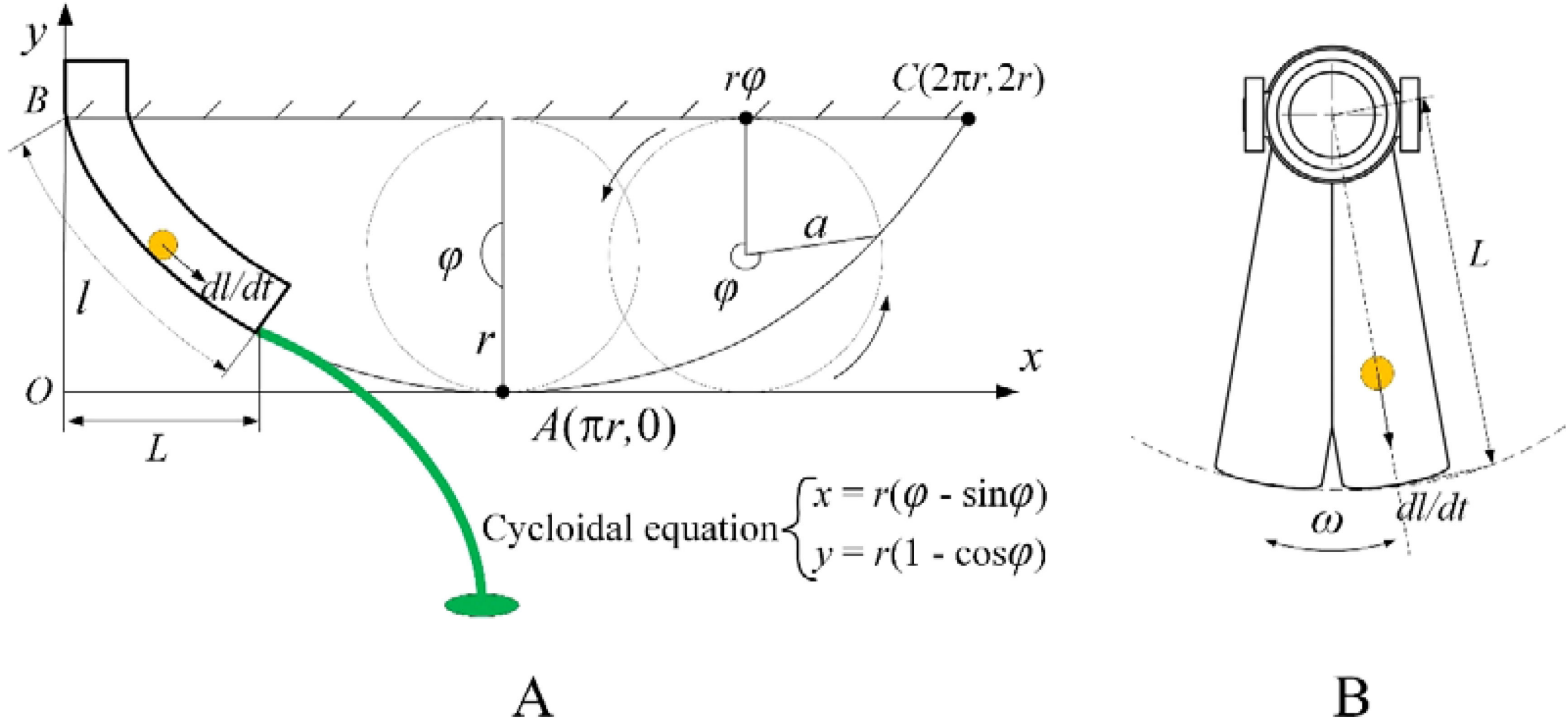
Velocity analysis schematic diagram. **(A)** Velocity analysis section view of the maximum velocity curve. **(B)** Velocity analysis plan for fertilizer particles in a tube.

The bifurcated swing tube fertilizer spreading device is made of fertilizer particles discharged from the fertilizer discharge opening by the fertilizer discharger, which naturally falls into the swing tube by gravity. Moreover, the swing tube adopts the principle of the most rapid curve design; therefore, it sprinkles the field in the form of a parabola from the mouth of the swing tube, as shown in **Figure 7A**, in which the actual length of the swing tube is set to *l*.

To investigate the equation of motion of the fertilizer particles, it is necessary to know the velocity at which the fertilizer particles leave the mouth of the swing tube, as shown in **Figure 7B**. When the bifurcated swing tube swings left and right with an angular velocity of *ω*, fertilizer particles move to the mouth of the swing tube; the velocity is synthesized to obtain the velocity *v* at this point, which is given by Equation 12.


(12) $$v=\sqrt{{\left(\frac{dl}{dt}\right)}^{2}+{\left(\omega L\right)}^{2}}$$

The length of the swing tube *l* is equal to the length of the pendulum line of a circle of radius *r* rolling at a certain angle *φ*. Knowing the cycloidal equation, the length of the cycloidal *l* can be found using the following equation.


(13) $$l=\int_{t=0}^{t=\varphi }\sqrt{{\left(\frac{dx}{dt}\right)}^{2}+{\left(\frac{dy}{dt}\right)}^{2}}dt$$

Substituting Equation 13 into Equation 12 yields:


(14) $$v=\sqrt{4a^{2}sin^{2}\frac{\theta }{2}+\omega^{2}L^{2}}$$

**Figure 8:**
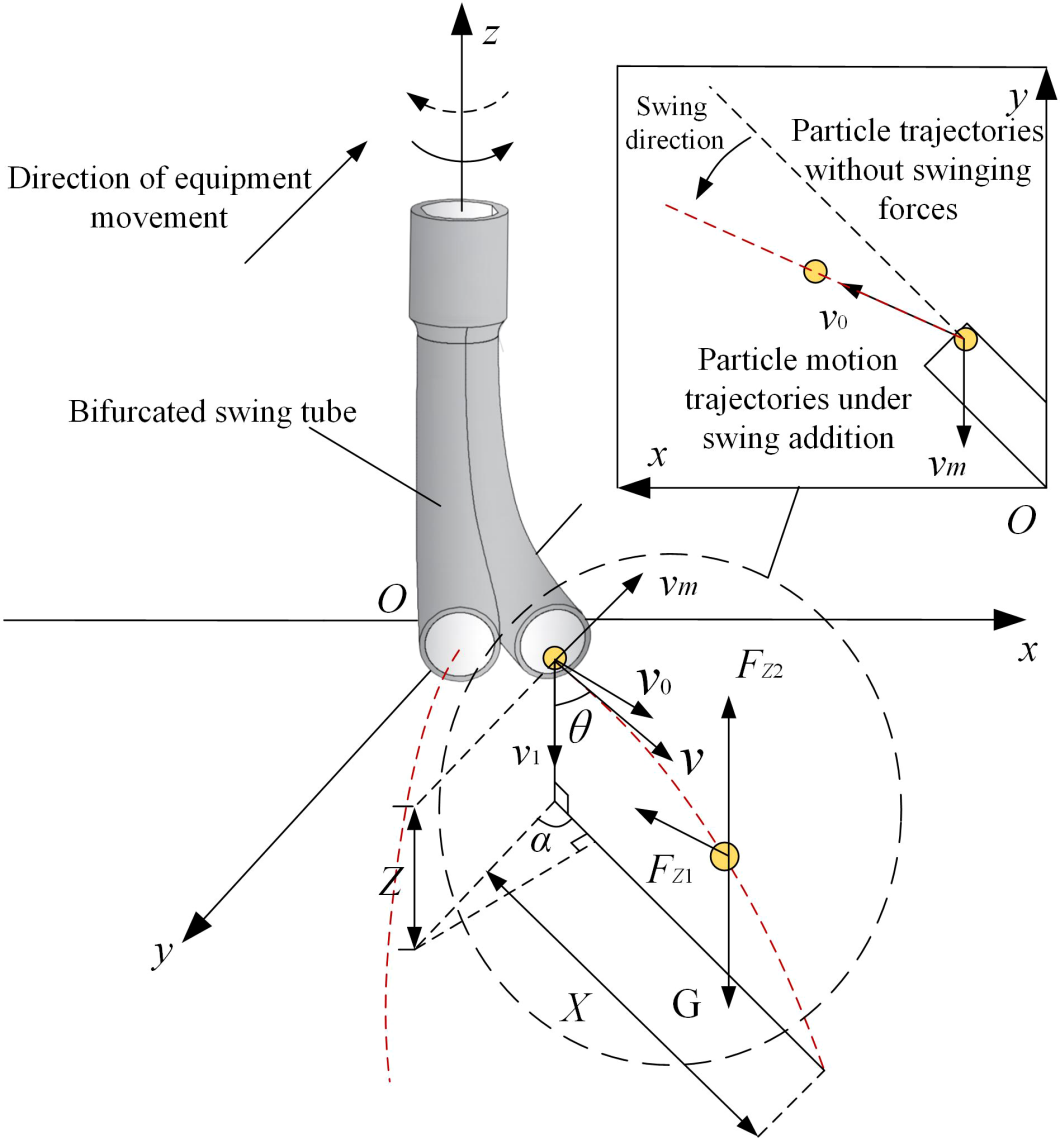
Motion and force analysis of fertilizer particles in air. Note: *v_m_* is the forward velocity of the device (m/s) and *Z* is the nozzle height (mm).

Because the fertilizer particles undergo parabolic motion with an initial velocity *v*, and when the throwing speed and the tube mouth height changes, the effect of spreading fertilizer also changes. Therefore, the horizontal and vertical distances of fertilizer particles thrown are studied, the initial velocity *v* is decomposed into plane velocity *v_0_* and vertical velocity *v_1_*, and the *oxyz* spatial right-angled coordinate system is established with the center of the bifurcated swing tube as the coordinate origin. The movement of fertilizer particles in the air and the forces applied to them are shown in **Figure 8**.

It is assumed that the self-rotation of the fertilizer particle and the airborne wind speed are ignored. Fertilizer particles moving in the air will be subjected to gravity and drag, and free fall along the direction of the nozzle tends to tilt the trajectory in the direction of the swing velocity. The force acting on the fertilizer particles in the air is shown by Equation 15 from the air resistance coefficient equation.


(15) $$F_{z}=\frac{1}{2}\cdot c\cdot s\cdot \rho_{a}\cdot v^{2}$$

where *F_z_* is the air resistance of the fertilizer particle during their movement (N), *c* is the coefficient of air resistance of the fertilizer particle and was determined to be *c* = 0.44 (Tian et al., 2023), *s* is the area of the wind resistance region of the fertilizer particle (m^2^), *ρ_a_* is the air density (kg/m^3^), and *v* is the fertilizer particle velocity (m/s).

According to Newton’s second theorem, the force balance equations for the movement of fertilizer particles in the plane and vertical directions are shown in Equations 16 and 17.


(16) $$\left\{ \begin{array}{l} m\frac{dv_{0}}{dt}=-F_{z1}\\ m\frac{dv_{1}}{dt}=G-F_{z2} \end{array}\right.$$

(17) $$\left\{ \begin{array}{l} F_{z1}=\frac{c\cdot s\cdot \rho_{a}}{2}\sqrt{v_{0}^{2}+v_{1}^{2}}\cdot v_{0}\\ F_{z2}=\frac{c\cdot s\cdot \rho_{a}}{2}\sqrt{v_{0}^{2}+v_{1}^{2}}\cdot v_{1}\\ G=mg \end{array}\right.$$

where *v*_0_ and *v*_1_ are the horizontal and vertical partial velocities of the fertilizer particle in motion, respectively (m/s), *Fz*_1_ and *Fz*_2_ are the horizontal and vertical components of the air resistance on the fertilizer particles during their movement respectively (N), $m=\frac{4\pi }{3}\rho_{p}\cdot L^{3}$ , *s* = π*L* ^2^, and *ρ_p_* is the density of the fertilizer particle (kg/m^3^).

The differential equation is formed from Equations 15 and 16.


(18) $$\left\{ \begin{array}{l} \frac{dv_{0}}{dt}=-\frac{3c\cdot \rho_{a}}{8\rho_{p}\cdot L}\cdot \sqrt{v_{0}^{2}+v_{1}^{2}}\cdot v_{0}\\ \frac{dv_{1}}{dt}=g-\frac{3c\cdot \rho_{a}}{8\rho_{p}\cdot L}\cdot \sqrt{v_{0}^{2}+v_{1}^{2}}\cdot v_{1} \end{array}\right.$$

Let $\frac{3c\cdot \rho_{a}}{8\rho_{p}\cdot L}=j$ be the scale factor. As the machine moves in the opposite direction to the direction of the fertilizer throw, *v*_0_ = *v*sin*θ* - *v_m_* cos*α* and *v*_1_ = *v*cos*θ*. Expressions for the distance *X* in the plane of the throw and the vertical fall distance *Z* can be derived.


(19) $$\left\{ \begin{array}{l} X=\left(vtsin\theta -v_{m}tcos\alpha \right)\left(1-\frac{jt}{2}\sqrt{v^{2}-2vsin\theta \cdot v_{m}cos\alpha +v_{m}^{2}cos^{2}\alpha }\right)\\ Z=vcos\theta \cdot t\left(1-\frac{jt}{2}\sqrt{v^{2}-2vsin\theta \cdot v_{m}cos\alpha +v_{m}^{2}cos^{2}\alpha }\right)+\frac{gt^{2}}{2} \end{array}\right.$$

where *θ* is the angle between the velocity of the fertilizer particle and the vertical component velocity (°), and *α* is the angle between the horizontal partial velocity of the fertilizer particle and the velocity of the device moving in the opposite direction (°).

Combining the above substitutions yields the trajectory equation for the fertilizer application process.


(20) $$Z=\frac{X\cdot \sqrt{4asin^{2}\frac{\theta }{2}+\omega^{2}L^{2}}\cdot cos\theta }{\sqrt{4a^{2}sin^{2}\frac{\theta }{2}+\omega^{2}L^{2}}\cdot sin\theta -v_{m}\cdot cos\alpha }+\frac{gt^{2}}{2}$$

From the above comprehensive analysis, it can be seen that when the proportionality coefficient *j* is specific, the landing position of the bifurcated fertilizer spreading device is mainly determined by the dynamic parameters of the nozzle height *Z*, the forward velocity *v_m_* of the device, and the angular velocity *ω*. The relationship of *ω* = 2π*f* is known. The subsequent study can be regarded as the swing tube frequency f as the influence of the dynamic parameters of the fertilizer spreading device on the fertilizer spreading effect. Therefore, in this study, the dynamic parameters of the fertilizer spreading device are firstly optimized through simulation tests by taking the nozzle height *Z*, the forward velocity *v_m_*, and the swing frequency *f* as the test factors and improving the uniformity of the fertilizer spreading effect as the optimization criterion. Finally, a bench test was conducted to verify whether optimizing the different dynamic parameters could improve the uniformity of fertilizer spreading.

## 4 MBD-DEM coupled simulation analysis

The dynamic parameters in the action of the fertilizer spreading device have a direct influence on the uniformity of fertilizer spreading, and to clarify the fertilizer spreading characteristics of the bifurcated swing tube, it is proposed that the discrete element method (DEM) will be used for the simulation. However, in fertilizer spreading by a bifurcated swing tube, the presence of a spatial hammer pendulum crank mechanism makes it difficult to realize the actual movement condition of the spatial mechanism in the working environment in EDEM. Therefore, a coupled multibody dynamics-discrete element (MBD-DEM) simulation is used for the analysis. Accurate results are obtained for the kinetic behavior of the particles in the system and the calculation of the forces acting on the particles in the dynamic system (Shi et al., 2022; Deng et al., 2024; Wang et al., 2021).

### 4.1 Multibody dynamics simulation establishment

**Figure 9:**
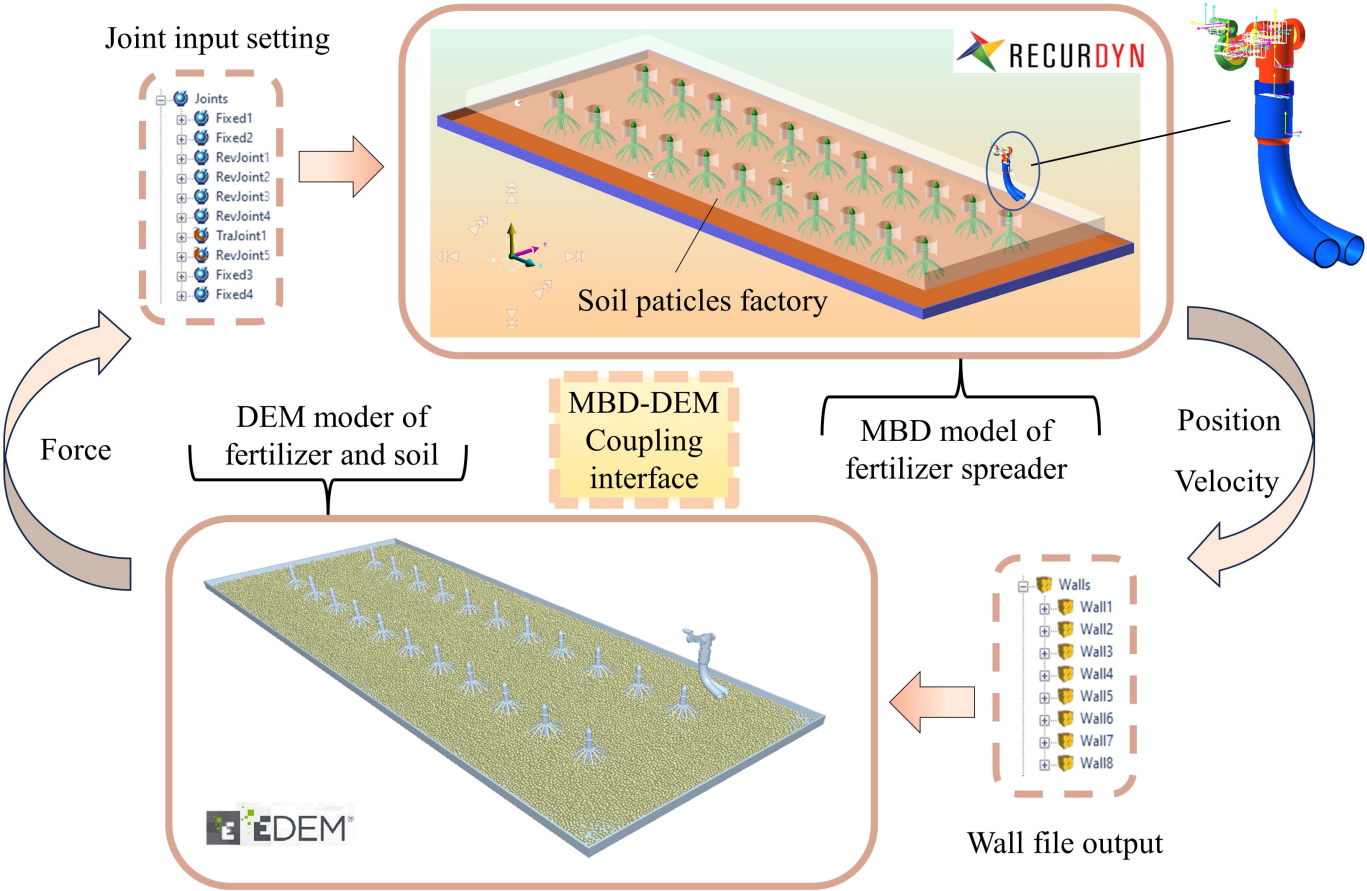
Schematic diagram of the MBD-DEM coupling device.

RecurDyn is suitable for solving large-scale complex multibody system dynamics problems. Owing to the complex motion of the bifurcated swing fertilizer spreading device, its simplified geometrical model is established through SolidWorks. An x-t format file is imported into RecurDyn and constraints, contacts, and other attributes are added to build a multi-body dynamics model. The components of the fertilizer spreading device were imported into EDEM as wall files through RecurDyn’s built-in coupling interface to create a discrete element model. The maize single-row planting standard is used as an example to simulate the field operation situation. The simulation model consists of a fertilizer spreading device, a fertilizer particles factory, crops, soil ground, and a soil particles factory. The distance between the crop rows is 600 mm, the nozzle height from the ground is 450.0 mm, the motor drive speed is 90 rpm (the swing tube swing frequency is 1.50 Hz), and the fertilizer spreading device moves with a velocity of 0.5 m/s. The velocity and position of the critical components are controlled by calculating Recurdyn’s equations of motion, and the data are transferred to EDEM through the coupled interface software Recurdyn for co-simulation to analyze the motion process of particle collision, as shown in **Figure 9**.

### 4.2 Multibody dynamics simulation establishment

The test fertilizer was selected from urea produced by the Shanxi Shanhua Coal Chemical Industry Group Co. The measured equivalent diameter averaged 1.16 mm, with a sphericity greater than 90%. As the fertilizer is thrown to the ground and comes into contact with the soil and crops during the actual spreading operation, the ground with soil particles is set up to give soil properties to restore the actual contact state of the fertilizer with the soil and crops. The Hertz–Mindlin with the JKR model was used for soil particles to soil particles (Li et al., 2023), whereas Hertz–Mindlin (no-slip) was used as a contact model for urea to urea, urea to the soil, and soil to crop (Zhao et al., 2023). Referring to the related literature (Dun et al., 2023b; Zhao et al., 2021b; Song et al., 2023b), the relevant parameter settings of the simulation model are shown in **Table 2**.

**Table 2:** Discrete element simulation parameter settings.

Project	Property	Value
	Poisson ratio	0.25
Urea	Shear modulus (Pa)	1.02×10^7^
	Density (kg·m^3^)	1345
	Poisson ratio	0.25
Soil	Shear modulus (Pa)	1.0×10^8^
	Density (kg·m^3^)	2000
	Poisson ratio	0.28
Fertilizer spreading device	Shear modulus (Pa)	8.0×10^9^
	Density (kg·m^3^)	1240
	Poisson ratio	0.33
Crops	Shear modulus (Pa)	6.39
	Density (kg·m^3^)	107.64
	Restitution coefficient	0.60
Urea-urea	Static friction coefficient	0.40
	Rolling friction coefficient	0.01
	Restitution coefficient	0.60
Urea-soil	Static friction coefficient	0.50
	Rolling friction coefficient	0.50
	Restitution coefficient	0.60
Urea-crops	Static friction coefficient	0.60
	Rolling friction coefficient	0.20
	Restitution coefficient	0.01
Urea-fertilizer spreading device	Static friction coefficient	0.02
	Rolling friction coefficient	0.01
	Restitution coefficient	0.60
Soil-crops	Static friction coefficient	0.60
	Rolling friction coefficient	0.02

### 4.3 Coupling simulation

**Figure 10:**
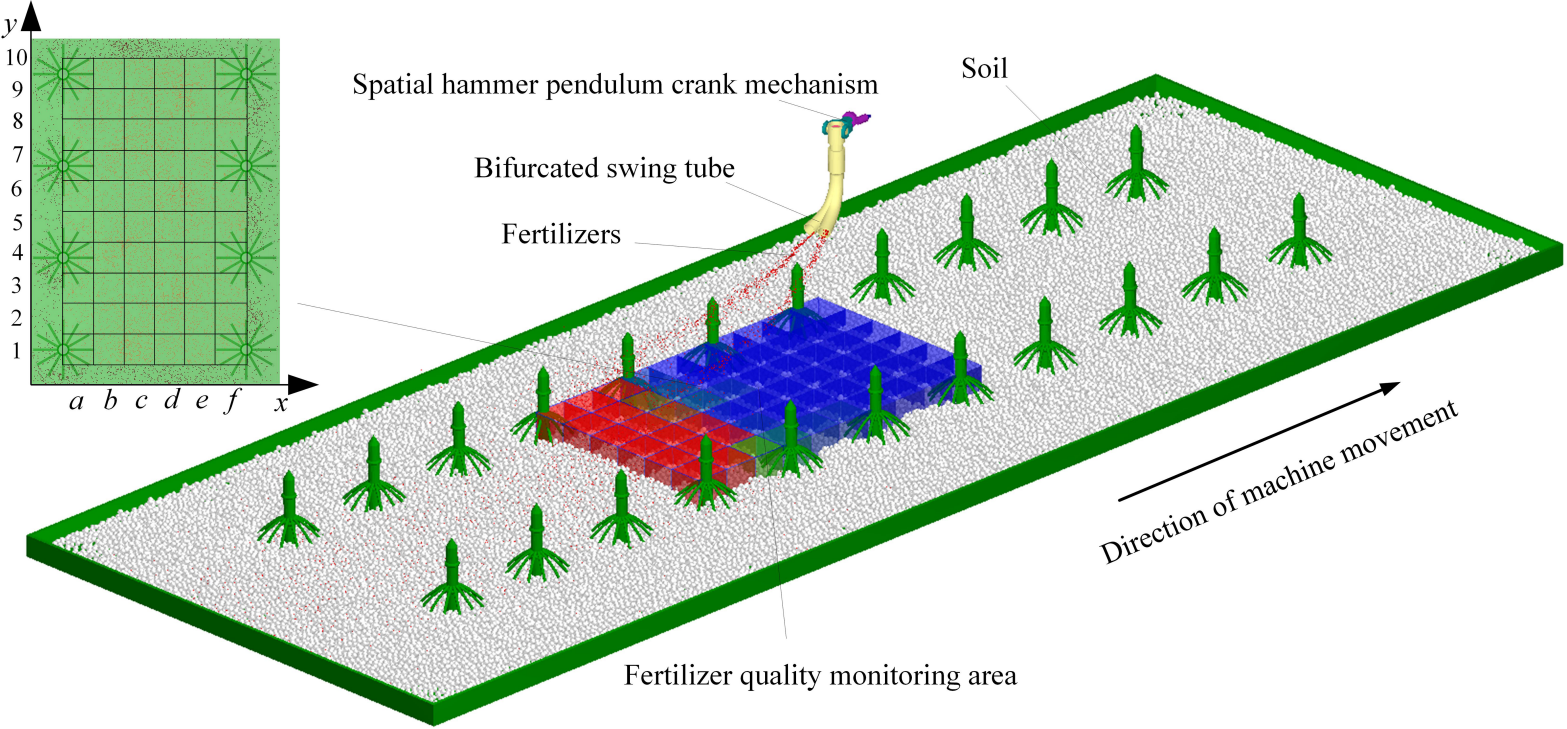
Coupled model effect diagram.

The swing tube fertilizer spreading device and the land components were imported into EDEM as a wall file through RecurDyn’s inbuilt coupling interface to create a discrete metamodel. The soil was simplified to spherical particles, set to a radius of 7 mm, and the soil particle factory was set to generate 90,000 soil particles so that they would naturally spill onto the land collection plate. When the soil is spread over the land collection plate, the fertilizer particles factory is synchronized with the bifurcated fertilizer spreading tube at equal velocity. The fertilizer pellet plant is set to generate 5,000 urea particles per second so that they are thrown to the soil surface with the swing of the bifurcated fertilizer spreading tube, and a fertilizer quality monitoring zone is set up at the soil surface, as shown in **Figure 10**.

### 4.4 Fertilizer spreading performance evaluation criteria

To accurately evaluate the uniformity of the bifurcated swing tube when spreading fertilizer, the performance was statistically and analytically analyzed using the grid method (Sugirbay et al., 2020; Wang et al., 2023b) at the end of the coupled simulation. The plant spacing direction *y* is defined as the longitudinal direction and the ridge spacing direction *x* as the transversal direction. As the fertilizer spreading device has the same movement pattern for each fertilizer spreading cycle, a fertilizer quality testing area with a length of 1,000 mm, a width of 600 mm, and a height of 300 mm for two cycles of fertilizer spreading is set up on the ground surface. Dividing six portions on *x* labeled with the letters *a* to *f* and ten portions on *y* labeled with the numbers 1 to 10 makes fertilizer quality monitoring in each cell possible.

Referring to the relevant literature (Zhang et al., 2018; Fan et al., 2024; Zhou et al., 2024), it is known that the evaluation index of fertilizer spreading uniformity is the uniformity coefficient. Fertilizer quality was selected for monitoring areas and measured for unit fertilizer quality monitoring simultaneously. The longitudinal and transverse homogeneity coefficients are calculated according to Equation 21, expressed as *Y*_1_ and *Y*_2_, respectively. The smaller the uniformity coefficient, the more uniformly the fertilizer is spread and *vice versa*.


(21) $$\left\{ \begin{array}{l} \sigma =\frac{S_{d}}{\bar{m}}\\ S_{d}=\sqrt{\frac{\sum_{i=1}^{n}{\left(m_{i}-\bar{m}\right)}^{2}}{n-1}}\\ \bar{m}=\frac{1}{n}\sum_{i=1}^{n}m_{i} \end{array}\right.$$

where *σ* is the uniformity coefficient of fertilizer spreading (%), *S_d_* is the standard deviation of the mass within the cell grid (g), $\bar{m}$ is the average mass within the grid of statistical cells (g), *m_i_* is the mass of fertilizer particles in the *i*th grid (g), and *n* is the number of statistical cells required.

## 5 Single-factor test

To determine the effects of nozzle height *Z*, forward velocity *v_m_*, and swing tube frequency *f* on the fertilizer spreading uniformity, a single-factor experimental study was carried out using an MBD-DEM coupled simulation under different factor variables, and Design-Expert was applied to carry out data processing and statistical analysis (Barbosa, 2020; Bai et al., 2021; Wang et al., 2024).

### 5.1 The effect of nozzle height *Z* on fertilizer spreading uniformity

**Figure 11:**
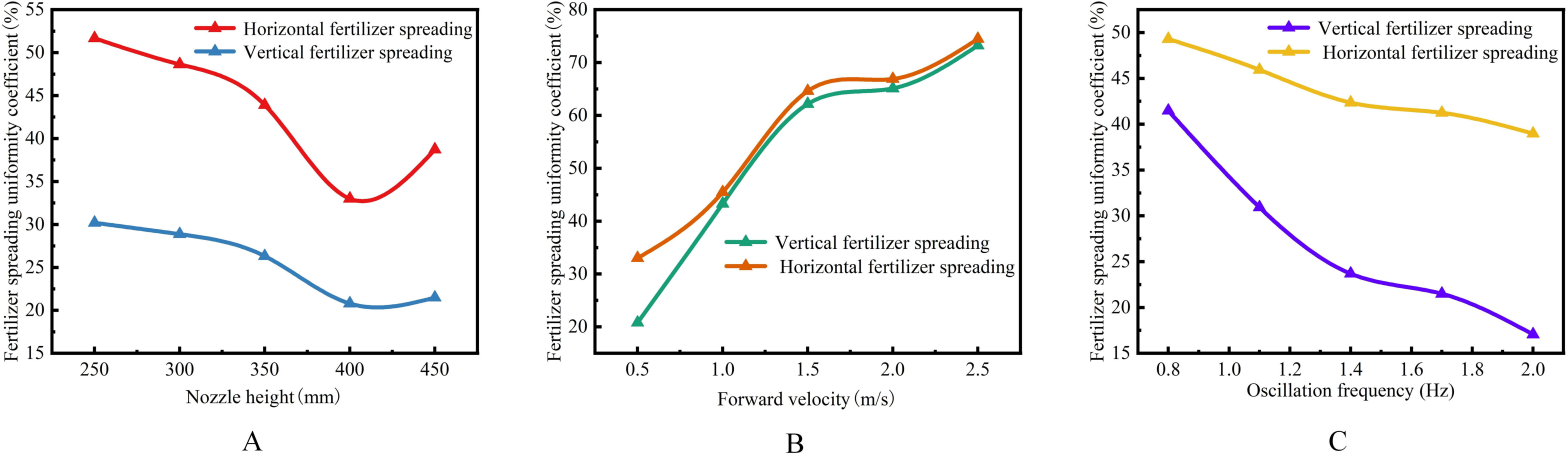
Impact curve of the single-factor test. **(A)** The effect of nozzle height *Z* on fertilizer spreading uniformity. **(B)** The effect of forward velocity *v_m_* on fertilizer spreading uniformity. **(C)** The effect of swing frequency *f* on fertilizer spreading uniformity.

The theoretical analysis shows that the nozzle height *Z* determines the spreading distance *X* and thus affects the spreading range *W*. At the same time, the longitudinal and transversal uniformity coefficients will also change with *Z*. Therefore, to investigate the effect of nozzle height *Z* on the uniformity of fertilizer spreading, a single-factor test was conducted with *Z* as the test factor and longitudinal and transversal uniformity coefficients as the test indexes. By reviewing the relevant literature (He et al., 2021; Zhao et al., 2021), the range of fertilizer spreading height *Z* was determined to be 250.0 mm < *Z* < 450.0 mm based on the height of the implement and agronomic characteristics and the design requirements of the fertilizer spreading machinery. Therefore, the test fixed swing tube pendulum frequency *f* = 1.50 Hz and the forward velocity of the device *v_m_* = 0.5 m/s selected the spreading of fertilizer height *Z* for 250.0 mm, 300.0 mm, 350.0 mm, 400.0 mm, and 450.0 mm for a single-factor test. The coefficient of longitudinal and transversal uniformity and the change trend are calculated in **Figure 11A**.

The longitudinal and transversal homogeneity coefficients showed a decreasing and then increasing trend with increasing height *Z*. A decreasing trend was observed before 400.0 mm, and an increasing trend was observed after 400.0 mm. The images show that after 350.0 mm, the trends of the two curves move differently, with a smaller uniformity coefficient indicating better fertilizer spreading performance. Therefore, 350.0 to 450.0 mm was selected to carry out further studies in conjunction with the effect of other factors on longitudinal and transversal uniformity.

### 5.2 The effect of machine forward velocity *v_m_* on fertilizer spreading uniformity

The bifurcated swing tube fertilizer spreading device moves on the suspension of the agricultural equipment, and the change of its forward velocity *v_m_* will change the initial velocity of the fertilizer throwing, thus affecting the size of the uniformity coefficient of the fertilizer spreading. If the device moves too fast, the fertilizer thrown per unit of time floats in the air for too long, and there are too many uncontrollable factors. The device also operates inefficiently at very low speeds; therefore, it was determined that the effect on the uniformity of fertilizer spreading should be investigated in the range of forward velocity 0.5 m/s < *v_m_* < 2.5 m/s. The forward velocities of 0.5 m/s, 1.0 m/s, 1.5 m/s, 2.0 m/s, and 2.5 m/s, the height of the swinging tube at a fixed distance from the ground *Z*=400.0 mm, and the frequency of swinging *f*=1.50 Hz were set to carry out a single-factor test on the effect of forward velocity *v_m_* on the uniformity of fertilizer spreading. The trends are shown in **Figure 11B**.

The uniformity coefficient of the longitudinal and transversal spreading of fertilizer increases with increasing forward velocity. The image at 1.5 m/s to 2.0 m/s slope is the smallest, adhering to the selection of a smaller uniformity coefficient as the best criterion for determining the range of values of the forward velocity of the machine in the range of 0.5 m/s to 2.0 m/s.

### 5.3 The effect of swing frequency *f* on fertilizer spreading uniformity

Based on the above theoretical analysis, the bifurcated swing tube swing frequency *f* affects the fertilizer spreading effect, hence the uniformity variation. As the spatial crank rotates around the input shaft for 1 week, the bifurcated swing tube swings back and forth from side to side for one cycle. Therefore, the drive speed is too fast, affecting the stability of the spatial hammer pendulum crank mechanism. If the speed is too low, then the bifurcated swing tube swing frequency is too low, and the process of moving implements leads to the swing cycle displacement being too long and cannot be applied to the crop in place. Therefore, a suitable swing frequency range of 0.80 to 2.00 Hz was taken to study the relationship with the uniformity of fertilizer spreading. The swinging frequency of the swing tube was set at 0.80 Hz, 1.10 Hz, 1.40 Hz, 1.70 Hz, and 2.00 Hz to carry out a comparative single-factor test of fertilizer spreading uniformity, in which the height of the limiting nozzle was 400.0 mm and the velocity of advancement was 0.5 m/s.

The trend is shown in **Figure 11C**, in which the overall uniformity coefficient of longitudinal and transversal fertilizer spreading decreases as the frequency of bifurcated swinging tube swinging increases simultaneously. Therefore, to take the condition that the uniformity coefficient of fertilizer spreading is minimized, it was determined that the swing frequency of the subsequent test was taken in the range of 1.40 to 2.00 Hz.

## 6 Response surface test

Based on the results of the above single-factor tests, a nozzle height range of 350.0 to 450.0 mm, a forward velocity of 0.5 to 1.5 m/s, and a swing frequency of 1.40 to 2.00 Hz were selected for the ternary-secondary universal rotary combination test conducted in this study. The factor level coding is provided in **Table 3**.

**Table 3:** Factor level coding table.

Code	Factor		
	Nozzle height *Z*/mm	Forward velocity *V_m_*/(m/s)	Swing frequency *f*/Hz
1.682	350.0	0.5	1.40
1	370.3	0.7	1.52
0	400.0	1.0	1.70
-1	429.7	1.3	1.88
-1.682	450.0	1.5	2.00

### 6.1 Test results and analyses

The experiment results are shown in **Table 4**, in which the factor-coded values are expressed as *x*_1_, *x*_2_, and *x*_3_, and the uniformity coefficients of longitudinal and transversal fertilizer spreading are expressed as _y1_ and *y*_2_, respectively.

**Table 4:** Test results.

Code	Test factor			Test indexes	
	Nozzle height *x*_1_	Forward velocity *x*_2_	Swing frequency *x*_3_	Longitudinal fertilizers spreading uniformity coefficient *y*_1_/%	Transversal fertilizers spreading uniformity coefficient y_2_/%
1	370.3	0.7	1.52	35.15	45.70
2	429.7	0.7	1.52	16.61	44.82
3	370.3	1.3	1.52	65.95	72.73
4	429.7	1.3	1.52	60.76	66.77
5	370.3	0.7	1.88	26.98	45.97
6	429.7	0.7	1.88	22.74	43.78
7	370.3	1.3	1.88	58.15	66.58
8	429.7	1.3	1.88	38.82	46.35
9	350.0	1.0	1.70	54.67	61.48
10	450.0	1.0	1.70	39.01	50.05
11	400.0	0.5	1.70	19.97	40.30
12	400.0	1.5	1.70	59.10	69.11
13	400.0	1.0	1.40	50.28	56.89
14	400.0	1.0	2.00	37.65	50.95
15	400.0	1.0	1.70	43.41	52.69
16	400.0	1.0	1.70	43.55	50.57
17	400.0	1.0	1.70	41.38	51.55
18	400.0	1.0	1.70	43.13	52.72
19	400.0	1.0	1.70	42.82	53.99
20	400.0	1.0	1.70	46.32	54.93

### 6.2 Variance analysis

Multiple regressions were fitted to the experimental data to obtain regression equations for longitudinal and transversal fertilizer spreading uniformity coefficients. The analysis of variance (ANOVA) of the model is shown in **Tables 5** and **6**. The regression model significance tests for longitudinal and transversal fertilizer spreading uniformity coefficients were highly significant (*P*<0.01). The results of the misfit term test for the uniformity coefficient of longitudinal fertilizer spreading were significant (0.01 < *P* < 0.05), and the results of the misfit term test for the uniformity coefficient of transversal fertilizer spreading had no effect (*P* > 0.1). This shows that both regression models fit well in the experimental range.

**Table 5:** ANOVA of the uniformity coefficient of longitudinal fertilizer spreading.

Evaluation indicator	Source of variance	Square Sum	DF	Mean square	*F*-value	*P*-value	Significance
Longitudinal fertilizers spreading uniformity coefficient *y*_1_/%	Model	3034.01	9	337.11	37.74	<0.0001	***
	*x*_1_	296.53	1	296.53	33.20	0.0002	***
	*x*_2_	2320.23	1	2320.23	259.76	<0.0001	***
	*x*_3_	290.82	1	290.82	32.56	0.0002	***
	*x*_1_ *x*_2_	17.23	1	17.23	1.93	0.1950	
	*x*_1_ *x*_3_	12.10	1	12.10	1.36	0.2714	
	*x*_2_ *x*_3_	39.16	1	39.16	4.38	0.0627	*
	*x*_1_^2^	11.07	1	11.07	1.24	0.2917	
	*x*_1_^2^	41.97	1	41.97	4.70	0.0554	*
	*x*_1_^2^	0.28	1	0.28	0.032	0.8622	
	Residual	89.32	10	8.93			
	Misfit term	76.29	5	15.26	5.85	0.0375	**
	Pure error	13.03	5	2.61			
	Total variation	3123.33	19				

*** *P* < 0.01 (Highly significant); **0.01 < *P* < 0.05 (Significant); *0.05 < *P* < 0.1 (Effect); *P* > 0.1 (Not effect).

**Table 6:** ANOVA of the uniformity coefficient of transversal fertilizer spreading.

Evaluation indicator	Source of variance	Square Sum	DF	Mean square	*F*-value	*P*-value	Significance
Transversal fertilizers spreading uniformity coefficient *y*_2_/%	Model	1534.09	9	170.45	37.34	<0.0001	***
	*x*_1_	172.12	1	172.12	37.71	0.0001	***
	*x*_2_	1065.21	1	1065.21	233.35	<0.0001	***
	*x*_3_	102.04	1	102.04	22.35	0.0008	***
	*x*_1_ *x*_2_	66.82	1	66.82	14.64	0.0033	***
	*x*_1_ *x*_3_	30.34	1	30.34	6.65	0.0275	**
	*x*_2_ *x*_3_	83.20	1	83.20	18.23	0.0016	***
	*x*_1_^2^	11.49	1	11.49	2.52	0.1437	
	*x*_1_^2^	3.87	1	3.87	0.85	0.3789	
	*x*_1_^2^	0.83	1	0.83	0.18	0.6780	
	Residual	45.65	10	4.56			
	Misfit term	33.16	5	6.63	2.66	0.1537	
	Pure error	12.49	5	2.50			
	Total variation	1579.74	19				

*** *P* < 0.01 (Highly significant); **0.01 < *P* < 0.05 (Significant); *0.05 < *P* < 0.1 (Effect); *P* > 0.1 (Not effect).

For the coefficient of longitudinal fertilizer spreading uniformity, *x*_1_, *x*_2_, and *x*_3_ had a highly significant effect on the equation (*P* < 0.01), *x*_2_ *x*_3_ and *x*_2_^2^ affected the equation (0.05 < *P* < 0.1), and the rest of the terms did not have an effect on the equation (*P* > 0.1).

For the uniformity coefficient of transversal fertilizer spreading, the effect of *x*_1_, *x*_2_, *x*_3_, *x*_1_ *x*_2_, and *x*_2_ *x*_3_ on the equation was highly significant (*P* < 0.01), the effect of *x*_1_ *x*_3_ on the equation was significant (0.01 < *P* < 0.05), and the rest of the terms had no effect on the equation (*P* > 0.1).

Removing the uninfluenced factors from the regression equation, the regression equation of the factors with the coefficients of uniformity of longitudinal and transverse fertilizer spreading can be known.


(22) $$\left\{ \begin{array}{l} y_{1}=15.88-0.16x_{1}+154.88x_{2}+15.85x_{3}-41.72x_{2}x_{3}-20.06x_{2}^{2}\\ y_{2}=-385.87+0.83x_{1}+263.87x_{2}+192.38x_{3}-0.33x_{1}x_{2}-0.37x_{1}x_{3}-60.81x_{2}x_{3} \end{array}\right.$$

### 6.3 Response surface analysis

**Figure 12:**
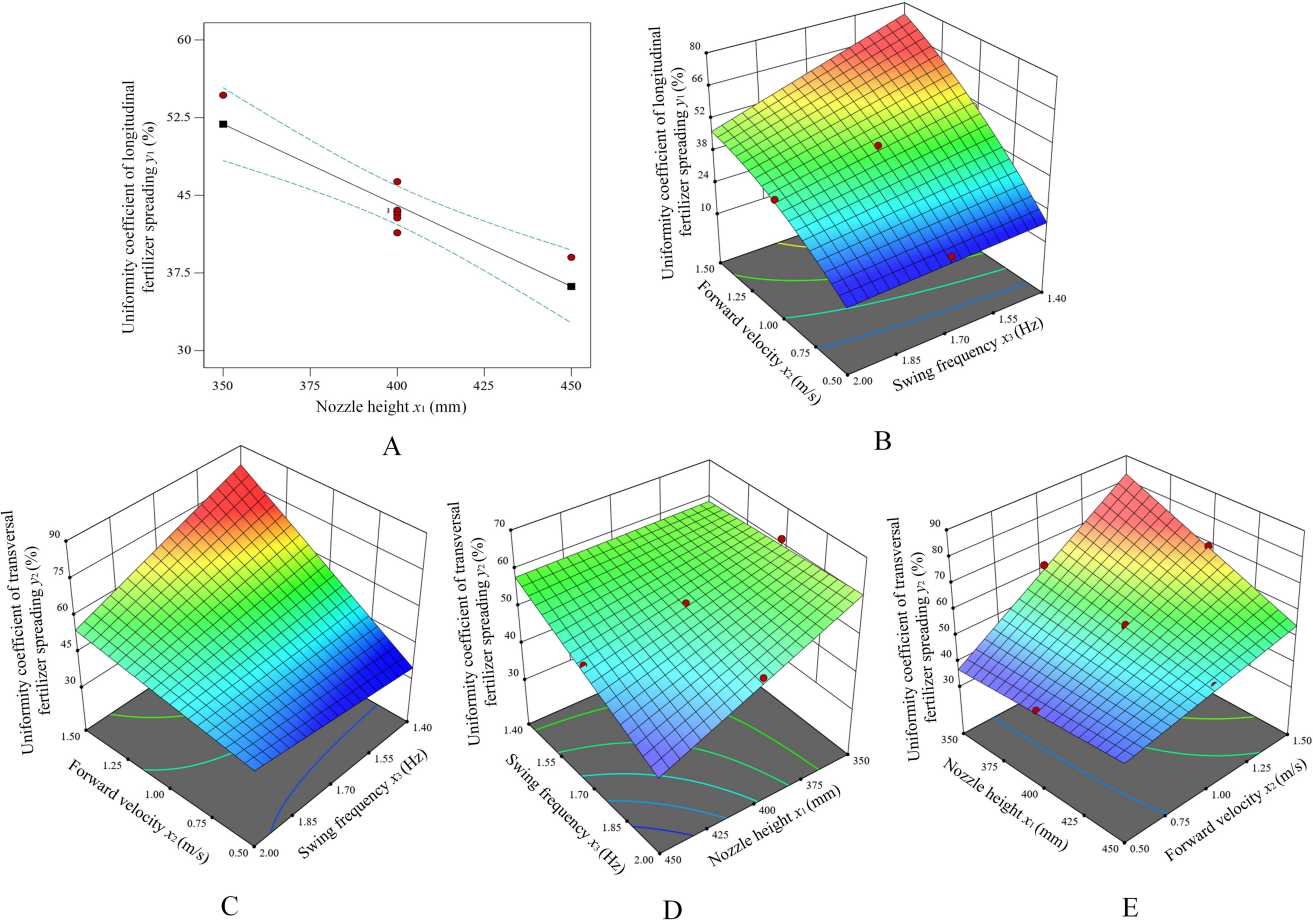
The effect of experimental factors on the uniformity coefficient of longitudinal and transversal fertilizer spreading. **(A)** The effect of *x*_1_ on *y*_1_. **(B)** The effect of the interaction of *x*_2_ and *x*_3_ on *y*_1_. **(C)** The effect of the interaction of *x*_2_ and *x*_3_ on *y*_2_. **(D)** The effect of the interaction of *x*_3_ and *x*_1_ on *y*_2_. **(E)** The effect of the interaction of *x*_1_ and *x*_2_ on *y*_2_.

The effect of each factor on the uniformity coefficient of fertilizer spreading in each longitudinal and transversal direction is shown in **Figure 12**. The nozzle height setting *x*_1_ for bifurcated swing tube fertilizer spreading is closely related to the longitudinal fertilizer spreading uniformity *y*_1_ coefficient, as shown in **Figure 12A**. It can be concluded that as *x*_1_ increases, *y*_1_ decreases gradually. Therefore, based on the maximum value of the nozzle height *x*_1_ = 450.0 mm, the response surface plot of the effect of the forward velocity *x*_2_ and the swing frequency *x*_3_ on the coefficient of longitudinal fertilizer spreading uniformity *y*_1_ is established, as shown in **Figure 12B**. As *x*_2_ increases, *y*_1_ increases significantly, and as *x*_3_ increases, *y*_1_ decreases. The *y*_1_ trend is highest at a low swing frequency and high speed because, in this case, the longitudinal distance of fertilizer application is too long when the swing tube swings at a unit angle, resulting in less coverage of the surface area passed by the fertilizer application path for the same swing amplitude, and less application over a long swing period. Under conditions of high swing frequency and low travel velocity, *y*_1_ tends to be lowest so that short swing periods with many application volumes occur, and fertilizer application paths can be multiplexed to cover the surface through which it passes.

Analysis of the response surface plot in **Figure 12C** shows that the uniformity coefficient of the lateral spreading of fertilizer *y*_2_ increases with the increase in the velocity of movement when *x*_3_ is low. *x*_2_ is low. The change in *x*_3_ does not have a significant effect on *y*_2_. The reason for this is that at high traverse velocities and low swing frequencies, the path of the fertilizer application does not effectively cover the surface through which it passes. Within the lateral width of the application per unit traveling distance, the amount of fertilizer at the extremes of the swing is low, resulting in a large number of “blank” unapplied areas and, therefore, an uneven lateral spreading of fertilizer. Analysis of **Figure 12D** reveals that *y*_2_​ has minimal impact on the trend observed when both *x*_1_​ and *x*_3_​ are set to high values. Moreover, the response surface plot tends to decline in the lowest parameter of *x*_1_ and *x*_3_, and *y*_2_ is minimum when *x*_1_ and *x*_3_ are minimum values. From the analysis in **Figure 12E**, it is clear that *x*_1_ is low and *y*_2_ increases as *x*_2_ increases. *x*_2_ is low, and the change in *x*_1_ is not significant for *y*_2_, as the uniformity of fertilizer spreading is better because the fertilizer is fully applied to the surface in low shift velocities, which increases the fertilizer coverage on the surface.

### 6.4 Parameter optimization

The uniformity coefficient of fertilizer spreading derived from the test should be based on the referenced NY/T1003-2006 Technical Specification for Quality Evaluation of Fertilizer Application Machinery (NY/T 1003-2006). To find the optimal combination of the relevant factors, it was determined that the nozzle height *x*_1_ = 450.0 mm, which gives the minimum value of the uniformity coefficient of longitudinal and transversal fertilizer spreading, should be the basis for optimizing the parameters. The smaller the uniformity coefficient of longitudinal and transversal spreading, the more uniformly the fertilizer is applied; therefore, the uniformity coefficient of longitudinal spreading is set to ≤25%, and the uniformity coefficient of transversal spreading is set to ≤45%. The optimization equations are shown in Equation 23 using Design-Expert software with multi-objective optimization (Liu et al., 2024; Wang et al., 2023) and the solved parameter ranges as constraints.


(23) $$\left\{ \begin{array}{l} x_{1}=450.0mm\\ 0.5\leq x_{2}\leq 1.5\\ 1.40\leq x_{3}\leq 2.00\\ y_{1}=f\left(x_{1},x_{2},x_{3}\right)\\ y_{2}=f\left(x_{1},x_{2},x_{3}\right)\\ y_{1}\leq 25\%\\ y_{2}\leq 45\% \end{array}\right.$$

**Figure 13:**
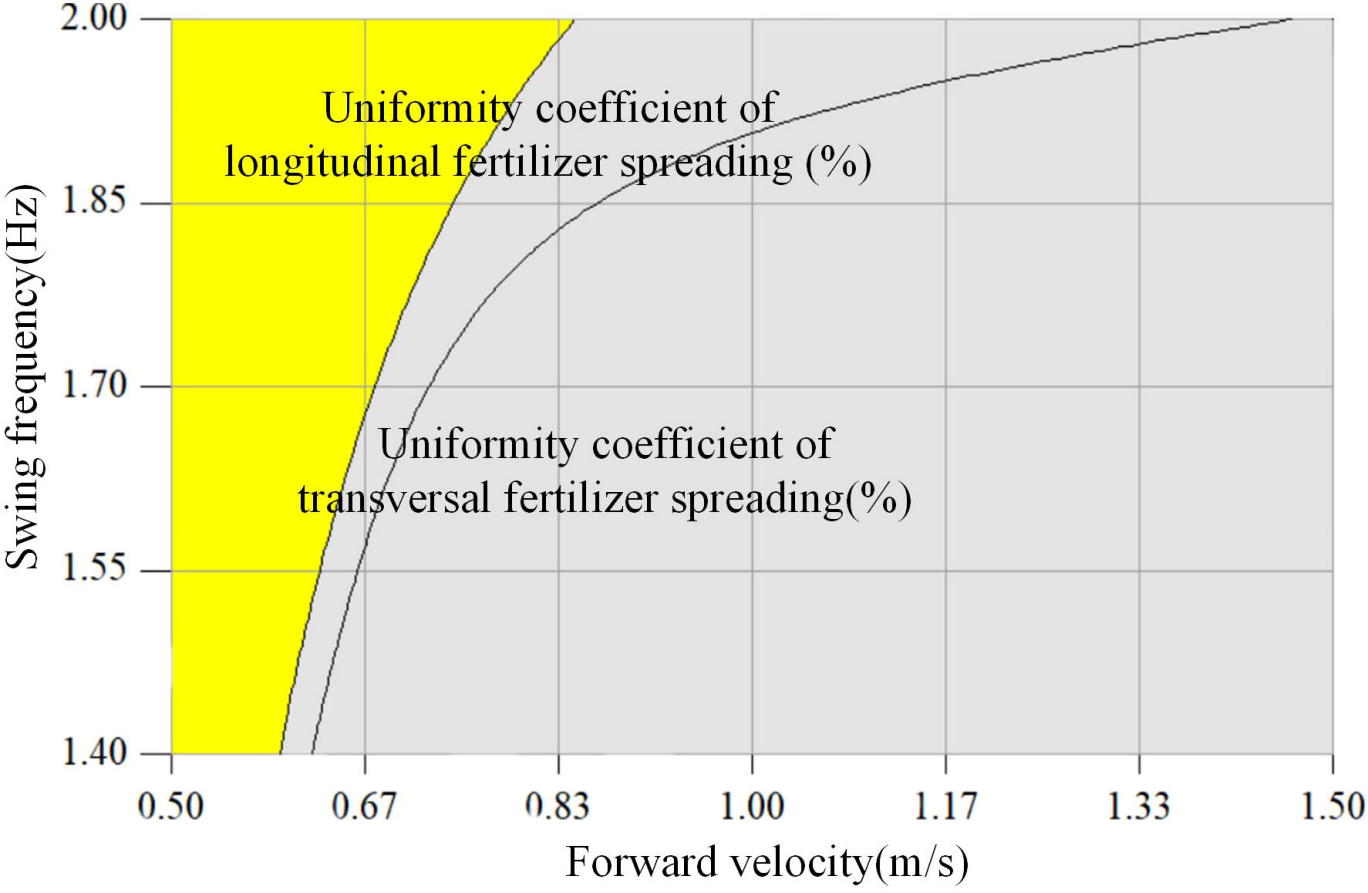
Parameter optimization.

Based on the above optimization Equation 23, the best optimization region for the forward velocity *x*_2_ and swing frequency *x*_3_ is obtained, as shown in the yellow shaded area in **Figure 13**. The optimized intervals are a forward velocity of 0.5–0.8 m/s and a swing frequency of 1.40–2.00 Hz.

The coupled simulation model was established to determine that the fertilizer spreading device meets the optimal fertilizer spreading effect by selecting the nozzle height *x*_1 =_ 450.0 _mm_, the forward velocity *x*_2 =_ 0.55 _m/s_, and the swinging frequency *x*_3 =_ 1.60 _Hz_. Simulation tests were carried out to verify the coefficients of the longitudinal and transversal fertilizer spreading uniformity of the dynamic parameters of the bifurcated swing tube fertilizer spreading device. The uniformity coefficients of longitudinal and transversal fertilizer spreading were obtained as 40.98% and 22.73% for the simulation validation values, respectively. The correctness of the optimization results is verified, and the data are analyzed below in comparison with the bench test data.

## 7 Bench test

### 7.1 Design of the experiment

To verify the correctness of the optimized parameters and the feasibility of the MBD-DEM coupled simulation verification experiment, the bifurcated swing tube fertilizer spreading device was machined and assembled, and the fertilizer spreading experiment platform was constructed. Bench experiments were conducted in May 2024 outside the Intelligent Agricultural Machinery and Equipment Engineering Laboratory at Harbin Cambridge College. According to the method of dividing the grid statistics of fertilizer distribution in the coupled simulation test, ten rows and six columns of 10 cm × 10 cm × 10 cm open square boxes are set up in the 60 cm × 100 cm test area with a total of 60 fertilizer collection points, to realistically restore the statistical area of the coupled simulation in the way of actual grid division. The fertilizer was selected from urea fertilizer produced by the Shaanxi Shanhua Coal Chemical Industry Group Co. The speed of the fertilizer discharge wheel of the fertilizer discharger used in the experiment was set at 60 r/min. The automatic lifting device controls the nozzle height from the ground at 450.0 mm, the swinging frequency of the swinging tube is 1.60 Hz, and the whole machine is towed by the power unit to control the forward velocity of the machine tool at 0.55 m/s. The control system selects the intelligent fertilizer controller produced by the Tripartite Agricultural Machinery Company, and the battery selects the 6-DZF-12 lead-acid battery model.

**Figure 14:**
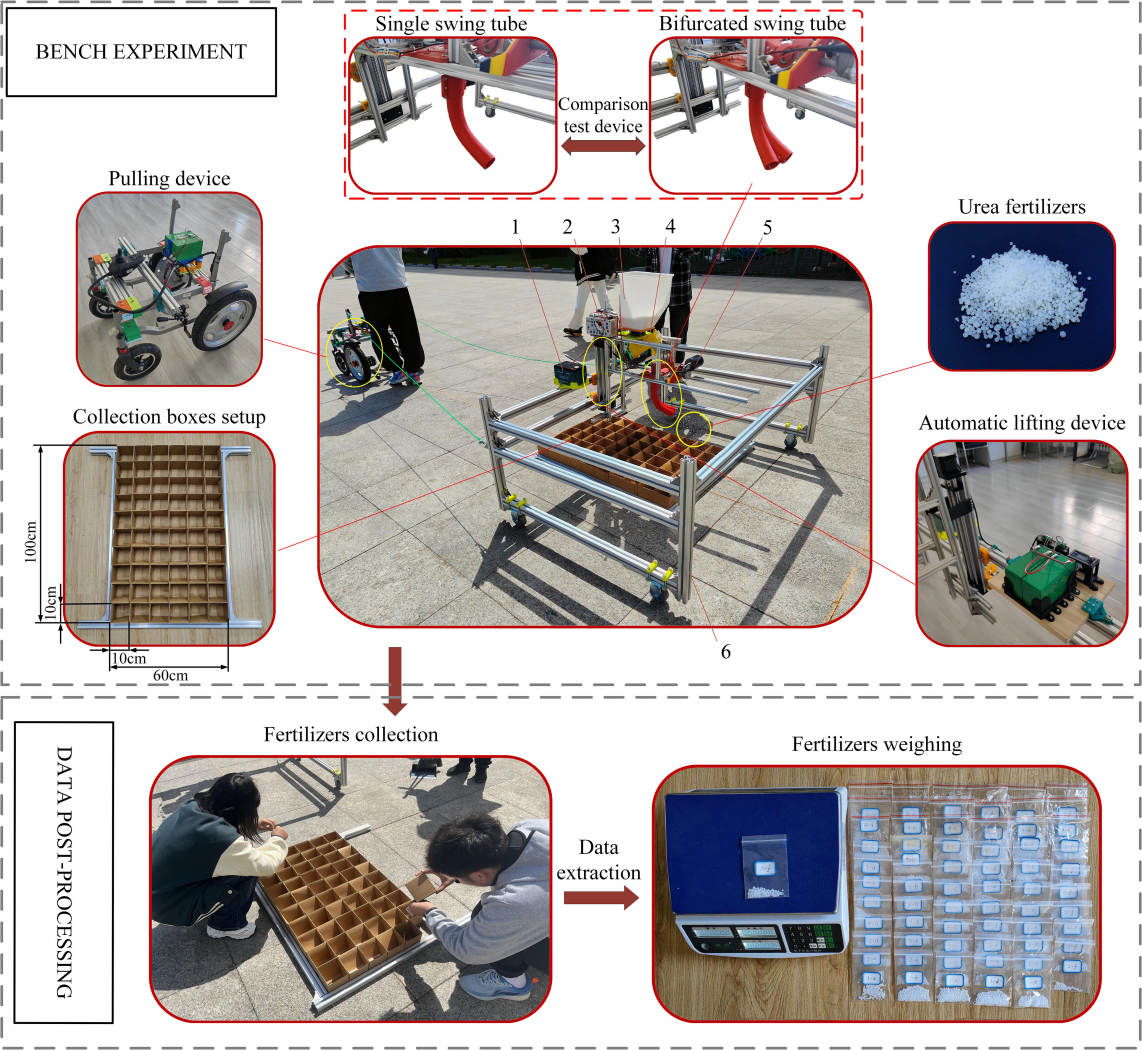
Bench validation and comparison experiment setup and post-processing. 1. Battery. 2. Motor controller. 3. Fertilizers tank. 4. Fertilizer discharger. 5. Drive motor. 6. Experimental bench.

Before the start of the experiment, the bench unit is positioned on the start line in the middle of the experiment boxes. After the bifurcated swing tube fertilizer spreading unit has been operated, the traction unit pulls the bench along the center line of the experiment grid. At the end of the experiment, the fertilizer in each collection box was filled up using collection bags. Once all the fertilizers were collected, the above experiment was repeated three times. Labeling each collection bag with collected fertilizer in the appropriate location makes it easy to count the results of the different location experiments. Each bag of experiment fertilizer was weighed with an electronic weighing scale and the husk was removed as it was weighed, as shown in **Figure 14**.

### 7.2 Comparative experiment

To further determine the fertilizer spreading advantages and disadvantages of a bifurcated swing tube over a single swing tube. Under the condition of the same nozzle height, swinging frequency and forward velocity, replace the single swing tube (the parameters of the single swing tube are the same as those of the bifurcated swing tube) to carry out the comparison test. The data from the simulation verification experiment and the comparison test were recorded, and the experiment results are shown in **Table 7**.

**Table 7:** Comparative validation results.

Fertilizer spreader tube type	Experimental methods	Experimental indicators	
		Longitudinal fertilizers spreading uniformity coefficient	Transversal fertilizers spreading uniformity coefficient
Single swing tube	Bench experiment	44.23%	47.50%
Bifurcated swing tube	Simulation experiment	22.73%	40.98%
	Bench experiment	21.94%	40.40%

By comparing the bench experiment data with the simulation data, the relative error of the longitudinal fertilizer spreading uniformity coefficient of the bench experiment was 3.46%, and the relative error of the transversal fertilizer spreading uniformity coefficient with the simulation experiment was 1.44%. The validation experiment was in general agreement with the simulation experiment, resulting in an error that could be due to the difference in the amount of fertilizer applied by the fertilizer discharger. By comparing the experimental data, the uniformity coefficient of the longitudinal fertilizer spreading of a bifurcated swing tube was reduced by 50.33%, and the uniformity coefficient of transversal fertilizer spreading was decreased by 14.95% compared with that of a single swing tube. The number of broken fertilizer particles in the bench experiment was minimal, and the broken particles were residual particles. The study can provide a reference for the optimal spiral fertilizer discharger design.

**Figure 15:**
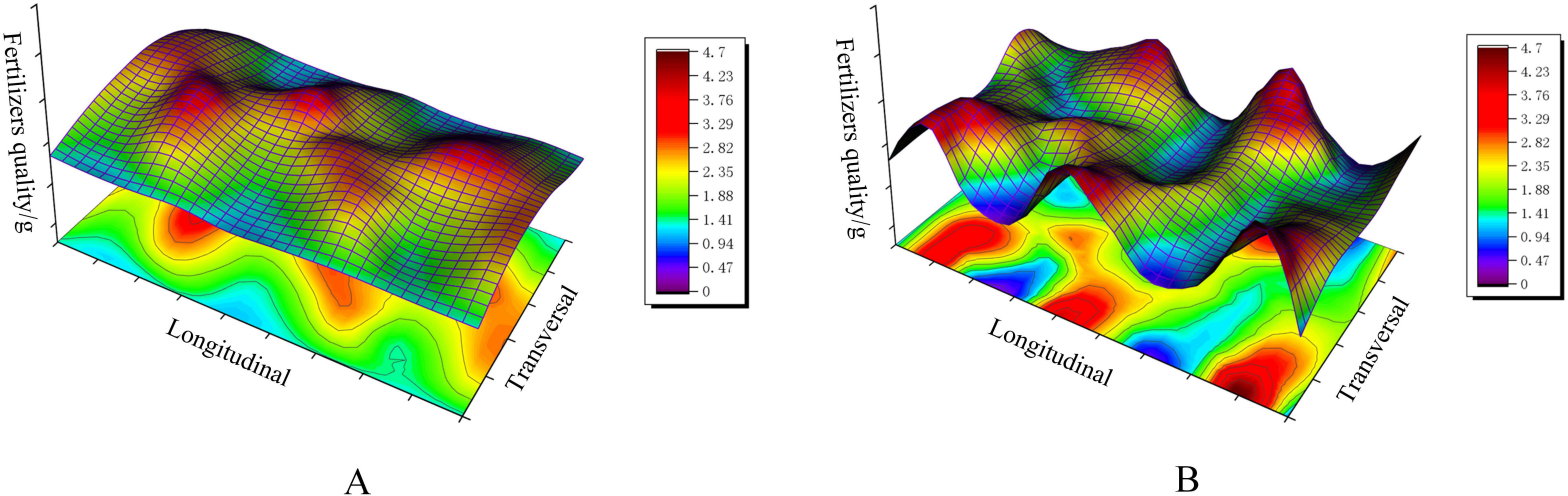
Fertilizer particle distribution with different swing tube spreaders. **(A)** Bifurcated swing tube fertilizer spreading distribution. **(B)** Single swing tube fertilizer spreading distribution.

To verify the reasonableness and accuracy of the test, a three-dimensional distribution map of overlapping fertilizer particles was plotted based on the bench comparison experiment data, as shown in **Figure 15**. It can be seen that the difference between the peak and the trough of the distribution of single swing fertilizer is significant, with some higher and lower sharp points, which is due to the effect of “one more, one less” staggered by the swing of the single swing tube structure. Although the bifurcated swing tube has a better fertilizer spreading distribution than the single swing tube, as can be seen from the contour map color, the dark blue color shows almost nothing, which can be proven to be a better fertilizer spreading balance in the unit area. The spreading uniformity coefficient of total fertilizer was calculated using Equation 21 and was 48.53% for a single swing tube and 38.12% for a bifurcated swing tube. It is proved that the bifurcated structure swing tube device spreads fertilizer evenly with good effect and meets the standard of fertilizer discharge performance of fertilizer application machinery specified in the referenced (NY/T1003-2006, 2015) Technical Specification for the Quality Evaluation of Fertilizer Application Machines (NY/T 1003-2006), which improves the utilization rate of fertilizers and promotes the sustainable development of agriculture. This study can be used as a theoretical reference for the design of a swing tube fertilizer spreading device.

## 8 Conclusion

1. In this study, to address the uneven spreading of fertilizer by a swing tube, a bifurcated swing tube fertilizer spreading device was designed based on the principle of the most rapid curve through the spatial hammer pendulum crank mechanism drive combined with the fertilizer discharger. The design of a spatial hammer pendulum crank mechanism based on a cylindrical pair achieves a change from a rotation to a swing, and the optimization of a bifurcated pendulum tube is based on the cycloidal equation. The influence of the dynamic parameters, such as the nozzle height, the forward velocity of the machine, and the swinging frequency of the swing tube, on the fertilizer spreading effect was theoretically analyzed, and the MBD-DEM coupled simulation model was established by using RecurDyn and EDEM.
2. The range of values of the parameters of each experiment factor was determined through a single-factor experiment, and a ternary two general-purpose rotary combination simulation experiment was designed using the uniformity coefficients of longitudinal and transversal fertilizer spreading as the experimental indexes. The relationship between the changes in the effects of the experiment’s factors on the experiment’s indicators was derived from the established regression models for the coefficients of longitudinal and transversal fertilizer spreading uniformity. The results showed that the effects of each experiment factor on each experiment index were highly significant and optimized to yield a longitudinal fertilizer spreading uniformity coefficient of less than 25% and a transversal fertilizer spreading uniformity coefficient of less than 45% when the nozzle height was 450.0 mm, the swing frequency was 1.50-2.00 Hz, and the operating velocity was within the range of 0.5-0.8 m/s.
3. Simulation and bench validation experiments were carried out under the optimal parameter combinations, and the experimental results agreed with the simulation results. The deviation values of the longitudinal and transversal uniformity coefficients were 1.44% and 3.46%, respectively, which were 50.33% lower than the uniformity coefficients of the longitudinal spreading of fertilizers by a single swinging tube and 14.95% lower than the uniformity coefficients of the transversal spreading of fertilizers. Moreover, the total uniformity coefficient of fertilizer spreading was 38.12%, which is in line with the (NY/T1003-2006, 2015) Technical Specification for the Quality Evaluation of Fertilizer Applying Machines, proving that the bifurcated swinging tube fertilizer spreading device can achieve the purpose of uniformly spreading fertilizers, improve the fertilizer utilization rate, and promote the sustainable development of agriculture.

## Data Availability

The raw data supporting the conclusions of this article will be made available by the authors, without undue reservation.
